# Recent Advances in Purple Sweet Potato Anthocyanins: Extraction, Isolation, Functional Properties and Applications in Biopolymer-Based Smart Packaging

**DOI:** 10.3390/foods13213485

**Published:** 2024-10-30

**Authors:** Dawei Yun, Yunlei Wu, Huimin Yong, Chao Tang, Dan Chen, Juan Kan, Jun Liu

**Affiliations:** College of Food Science and Engineering, Yangzhou University, Yangzhou 225127, China; dx120220228@stu.yzu.edu.cn (D.Y.); mz120232165@stu.yzu.edu.cn (Y.W.); 008694@yzu.edu.cn (H.Y.); 008155@yzu.edu.cn (C.T.); dchen@yzu.edu.cn (D.C.); kanjuan@yzu.edu.cn (J.K.)

**Keywords:** active packaging, anthocyanins, intelligent packaging, meat products, purple sweet potato

## Abstract

Petroleum-based plastic packaging materials have negative impacts on the environment and food safety. Natural biopolymer-based food packaging materials are the proper substitutes for plastic-based ones, which is because biopolymers are nontoxic, biodegradable and even edible. The incorporation of bioactive and functional substances into a biopolymer-based film matrix can produce novel smart packaging materials. Anthocyanins, one class of natural colorants with potent antioxidant activity and pH-response color-changing ability, are suitable for producing biopolymer-based smart packaging films. The purple sweet potato is a functional food rich in anthocyanins. In the past decade, numerous studies have reported the extraction of anthocyanins from purple sweet potato and the utilization of purple sweet potato anthocyanins (PSPAs) in biopolymer-based smart packaging film production. However, no specific review has summarized the recent advances on biopolymer-based smart packaging films containing PSPAs. Therefore, in this review, we aim to systematically summarize the progress on the extraction, isolation, characterization, purification and functional properties of PSPAs. Moreover, we thoroughly introduce the preparation methods, physical properties, antioxidant and antimicrobial activity, pH sensitivity, stability and applications of biopolymer-based smart packaging films containing PSPAs. Factors affecting the extraction and functional properties of PSPAs as well as the properties of biopolymer-based films containing PSPAs are discussed.

## 1. Introduction

The shelf life of foods is limited by several intrinsic and extrinsic factors, such as nutrients, water activity, temperature, relative humidity, illumination, oxygen, microorganisms, etc. Packaging is an effective practice for food preservation by providing protective barriers and reducing security risks [1]. Petroleum-based plastics, such as polyethylene, polypropylene, polyethylene terephthalate, polystyrene and polyvinyl chloride, are traditionally used in food packaging [2]. However, these plastic-based packaging materials have certain risks of environmental pollution and food safety. Thus, a new generation of food packaging materials originating from natural biopolymers is emerging, which is based on the nontoxic, biodegradable and even edible characteristics of biopolymers [3]. Moreover, biopolymers can act as the solid supports for a number of bioactive and functional substances, producing novel active packaging and intelligent packaging materials [4].

Active packaging and intelligent packaging, combined to form smart packaging, are two different techniques in modern food packaging [5,6]. Active packaging contains active substances, such as moisture/ethylene/oxygen scavengers, carbon dioxide emitters, antioxidant agents and antibacterial agents, that can extend the shelf life of foods [7]. Intelligent packaging incorporates functional substances, such as enzymes, antibiotics, sensors and indicators, that can trace and share the real-time information of food quality [8,9]. In recent years, researchers have found some natural colorants, such as anthocyanins, betacyanins, shikonin and curcumin, have antioxidant activity, antibacterial activity and color-changing ability in response to environmental pH changes [10,11,12]. Notably, the antioxidant and antibacterial activity of natural colorants can be used for food preservation, while the color-changing ability of the colorants can be utilized to indicate the freshness/spoilage degree of foods. Therefore, natural colorants can be added into biopolymer-based film matrix to fabricate smart packaging materials.

Anthocyanins, one class of natural colorants, have received the most attention in smart packaging production due to the rich sources, fast pH response and color-changing ability of anthocyanins [13,14]. Anthocyanins are widely distributed in plants, making plants show a wide array of colors, such as red, purple and blue. The color of anthocyanins originates from the mother nucleus, i.e., anthocyanidins [15]. Structurally speaking, anthocyanins are the glycosylated and acylated products of anthocyanidins [16]. In general, the structure and composition of anthocyanins from different plants vary a lot [14]. Thus, the biopolymer-based films incorporating anthocyanins from different plants normally show different performances in smart packaging [17].

Purple sweet potato (PSP) is a crop with rich nutrition and health benefits. The leaf, stem and root of PSP contain several bioactive substances, such as polysaccharides, proteins, anthocyanins and phenolic acids [18,19,20]. Among them, anthocyanins contribute to the purple color of PSP. In recent years, anthocyanins have been extracted from different cultivars of PSP by different means and characterized for their structures by using modern instrumental analysis methods [21]. Moreover, the obtained purple sweet potato anthocyanins (PSPAs) are found to possess good antioxidant activity and pH-response color-changing ability [22,23]. In this respect, a number of novel smart packaging films have been developed by adding PSPAs into a biopolymer-based film matrix. Despite this, no specific review has summarized the recent advances on the smart packaging films containing PSPAs. Therefore, in this review, the extraction, purification and properties of PSPAs as well as their applications in smart packaging are summarized and discussed.

## 2. Extraction of PSPAs

Until now, different means including solvent extraction, ultrasound-assisted extraction, enzyme-assisted extraction, microwave-assisted extraction, pulsed electric field extraction, pressurized liquid extraction, high-pressure carbon dioxide extraction and their combinations have been explored for the extraction of anthocyanins from the roots or leaves of PSP. No matter what kind of extraction method is selected, the raw material of PSP is normally washed to remove soil and dirt and cut into small sizes. In some circumstances, the root of PSP is even pre-cooked before the extraction of PSPAs [24,25,26,27,28]. The extraction efficiency of PSPAs is normally evaluated by measuring total anthocyanin content in the extract [29].

### 2.1. Solvent Extraction

**Figure 1:**
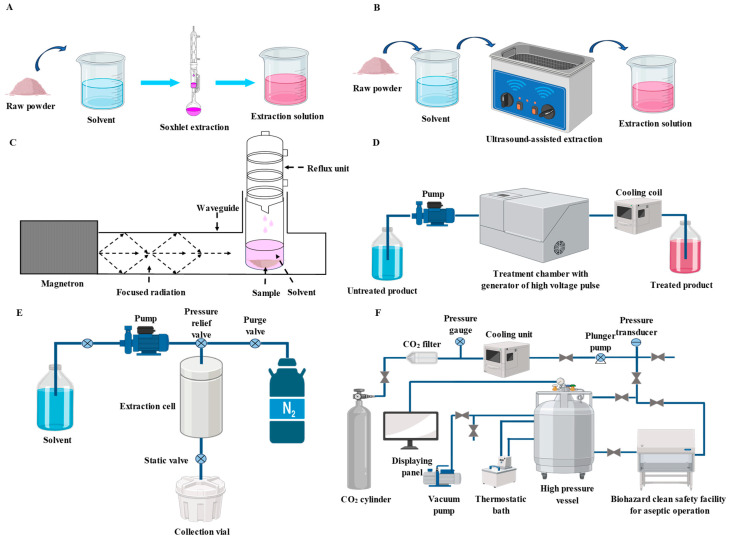
Different extraction methods of PSPAs: solvent extraction (**A**), ultrasound-assisted extraction (**B**), microwave-assisted extraction (**C**), pulsed electric field extraction (**D**), pressurized liquid extraction (**E**) and high-pressure carbon dioxide extraction (**F**).

Solvent extraction, due to its easy operation, has been most widely used in the extraction of PSPAs. As presented in Figure 1A, solvent extraction can be realized through maceration or a soxhlet extraction apparatus [30]. Acidified water/methanol and water/ethanol mixtures are usually selected as the extraction solvents for PSPAs. The acids (e.g., hydrochloric acid, acetic acid and citric acid) in the extractants plays an important role in the stabilization of PSPAs, which is because anthocyanins are stable in acidic solvents. Notably, the concentration of acids must be seriously controlled because strong acids can hydrolyze the glycosidic bond within PSPAs [27,31]. Except for acids, other solvents (e.g., water, methanol and ethanol) in the extractants have good solubility towards PSPAs with strong polarity. Interestingly, the unconventional solvents, such as aqueous two-phase [32], acidified acetone–butanol–ethanol mixture [33], acidic electrolyzed water [34] and polyethylene glycol [35] are sometimes considered for the extraction of PSPAs. Nonetheless, the removal of these unconventional solvents during the subsequent isolation and purification processes could be a big challenge. By contrast, the conventionally used solvents (e.g., acids, water, methanol and ethanol) can be easily removed from the extract through rotary evaporation. Notably, the solid–liquid ratio, solvent composition, extraction temperature and extraction time are the key factors affecting the extraction of PSPAs [36,37]. Some researchers have optimized the extraction conditions of PSPAs by different statistical analysis methods, such as full factorial experimental design [38], central composite design [39], Box–Behnken design [32,40,41] and face-centered cube design [42]. The optimal extraction conditions of PSPAs are summarized in Table 1. Although solvent extraction has been most widely used in the extraction of PSPAs, the main drawbacks of solvent extraction cannot be ignored, especially its low efficiency and harm to the environment. A new trend is to use more efficient and environmentally friendly solvents, such as deep eutectic solvents, to extract anthocyanins from plants, which should be deeply investigated in future [43].

### 2.2. Ultrasound-Assisted Extraction

Ultrasound-assisted extraction, due to its high efficiency, inexpensive equipment, and low solvent and energy requirements, has been widely applied in the extraction of PSPAs [35,37,44,50,51]. The ultrasound-assisted extraction of PSPAs involves several mechanisms, including erosion, fragmentation, sonoporation and sonocapillary effects [52]. In general, different kinds of ultrasound apparatus including ultrasonic bath, ultrasound reactor, ultrasound probe and continuous ultrasound equipment can be utilized in the extraction of PSPAs [52]. However, PSPAs are mainly extracted by using ultrasonic bath in existing studies (Figure 1B), where the ultrasonic power, ultrasonic frequency and ultrasonic intensity are normally uncontrolled [37,44,45]. As displayed in Table 1, the ultrasound-assisted extraction conditions of PSPAs have been optimized by several researchers using statistical analysis methods, such as Box–Behnken design [44], central composite design [46,51] and Taguchi orthogonal design [29]. Results show that the solid–liquid ratio, solvent composition, ultrasound temperature and ultrasound time can greatly influence the extraction yield of PSPAs [29,44,45,46,51]. In future, the impacts of ultrasonic power, ultrasonic frequency and ultrasonic intensity on the extraction of PSPAs could be further investigated by using other types of ultrasound apparatus. Ultrasound-assisted extraction in combination with aqueous two-phase extraction [51] and enzyme-assisted extraction [45,47] is a good choice to achieve higher extraction yield and extraction efficiency.

### 2.3. Enzyme-Assisted Extraction

Enzyme-assisted extraction is sometimes utilized for the extraction of PSPAs [45,47], where enzymes such as cellulase, pectinase, papain, amylase and glucoamylase are used to hydrolyze unwanted substances (e.g., cellulose, pectin, protein and starch) in PSP. However, the efficiency of enzyme-assisted extraction is very low, and thus enzyme-assisted and ultrasound-assisted extraction methods are normally used in combination [45,47]. Notably, the enzymes can be added into the extractants at different stages. Cellulase was added into the extractant and further subjected to ultrasound treatment, where the enzyme-assisted extraction and ultrasound-assisted extraction occurred simultaneously [45]. Compound enzymes containing cellulase, pectinase and papain were used to hydrolyze cellulose, pectin and protein in PSP. Afterwards, the enzymatic hydrolysate was subjected to ultrasound-assisted extraction [47]. As shown in Table 1, the extraction conditions for ultrasound–enzyme combined extraction for PSPAs have been optimized by Box–Behnken design [45,47]. Results show that the solid–liquid ratio, solvent composition, enzyme dosage, pH, enzymatic hydrolysis time, enzymatic hydrolysis temperature, ultrasound temperature and ultrasound time are important factors affecting the extraction yield of PSPAs [45,47]. In future, other enzymes such as hemicellulase, ligninase, proteases and pectin lyase could be tested for their suitability for the extraction of PSPAs.

### 2.4. Microwave-Assisted Extraction

Microwave-assisted extraction is based on the localized heating mechanism of microwave [53]. However, PSPAs are seldom extracted by using a microwave-assisted technique [39,48]. As displayed in Figure 1C, microwave-assisted extraction can be performed in an ordinary household microwave oven by macerating PSP in the extractant [39]. Microwave-assisted extraction can also be combined with other extraction techniques to extract PSPAs [39]. A soxhlet-like extractor containing vaporization and condensation units was connected to a microwave apparatus, and the improved microwave-assisted extraction apparatus showed a high extraction efficiency for PSPAs [48]. As summarized in Table 1, the extraction conditions of microwave-assisted extraction for PSPAs have been optimized by central composite design [39] and Box–Behnken design [48]. Results show that the solid–liquid ratio, solvent composition, pH, microwave power and microwave time can affect the extraction yield of PSPAs [39,48].

### 2.5. Pulsed Electric Field Extraction

Pulsed electric field is an environmentally friendly and minimally invasive technology to extract anthocyanins from plant matrices [54]. The application of pulsed electric field in the extraction of anthocyanins is mainly based on the electroporation effect, where the permeability of the cytomembrane and the mass transfer of intracellular components are increased under electric field [54]. Nonetheless, pulsed electric field technology is restricted by its high equipment cost and limited effectiveness (Figure 1D). Therefore, pulsed electric field has seldom been used in the extraction of PSPAs [55]. Recently, Bernabeu et al. [55] applied pulsed electric field technology to extract anthocyanins from PSP peels, and found that many other substances such as carbohydrates, minerals and polyphenols were also recovered from the raw material. Notably, the extraction yield of PSPAs might be influenced by electric field strength, pulse frequency and duration. However, the extraction conditions of PSPAs by pulsed electric field have not been optimized up to now.

### 2.6. Pressurized Liquid Extraction

Pressurized liquid extraction is an effective technique to extract bioactive compounds from plants by employing a solvent at an elevated temperature (50–150 °C) and pressure (3–21 MPa) [56]. Pressurized liquid extraction is normally performed in special equipment, where the temperature and pressure of the solvent are elevated (Figure 1E). In the extractor, dispersants such as sand and diatomaceous earth are added to reduce solvent channeling and particle clumping. The advantages of pressurized liquid extraction include the reduction of solvent used and extraction time. Despite this, this technique is restricted by its high equipment cost. Until now, only a few studies have applied pressurized liquid extraction for PSPAs [27,29,49]. The extraction conditions for extracting PSPAs by pressurized liquid have been optimized by face-centered cube design [49] and Taguchi orthogonal design [29], showing that the extraction temperature, solvent composition and solid–liquid ratio are the factors affecting the yield of PSPAs. In future, other potential factors (e.g., extraction pressure, pH and dispersant type) affecting the extraction yield of PSPAs could be further evaluated.

### 2.7. High-Pressure Carbon Dioxide Extraction

High-pressure carbon dioxide extraction is a green and non-thermal technique to extract bioactive substances from plants under high pressure (5–50 MPa) and mild temperature (5–80 °C), where high-pressure carbon dioxide can facilitate the recovery of targets by disrupting the cytomembrane, accelerating mass transfer and causing intracellular electrolyte balance disorder [57]. So high-pressure carbon dioxide can make the extraction process become easier and more efficient. Similarly to pressurized liquid extraction, high-pressure carbon dioxide extraction requires specific equipment, which contains a high-pressure vessel with accessory pressure and temperature controllers (Figure 1F). Until now, only one study has investigated the high-pressure carbon dioxide extraction of PSPAs [58]. Unfortunately, the extraction conditions of PSPAs by high-pressure carbon dioxide have not been investigated yet. In future, the potential factors (e.g., extraction temperature, extraction pressure and solid–liquid ratio) affecting the extraction yield of PSPAs could be tested. Moreover, supercritical carbon dioxide, a promising fluid generated at a temperature lower than 31.1 °C and pressure higher than 7.38 MPa [59], could be utilized in the extraction of PSPAs.

It can be concluded that the extraction yield of PSPAs is greatly influenced by extraction conditions. Among different extraction conditions, solvent type is an important factor affecting the extraction yield of PSPAs. For instance, Vishnu et al. [31] compared the yield of PSPAs in different extraction solvents and found methanol/TFA (99.5/0.5) was the most efficient solvent for extracting PSPAs. Zuleta-Correa et al. [33] investigated the effect of different organic solvents (i.e., methanol, ethanol, butanol and acetone) and their mixtures on the extraction of PSPAs, and they found the mixture of acetone, ethanol and butanol was more suitable for exacting PSPAs. Lao et al. [58] documented that PSPAs were more effectively extracted in the acidified 80% aqueous methanol than in the acidified water. Huang et al. [35] found the type and concentration of PEG had big impacts on the extraction yield of PSPAs, with PSP extracted in 80% PEG 200 having the highest yield of PSPAs. However, Lu et al. [34] reported that PSP extracted in 95% ethanol and acidified electrolyzed water had a similar yield of PSPAs.

Up until now, several researchers have compared the extraction yield of PSPAs obtained by different extraction methods [29,37,44,48,55,58]. Results show that the emerging extraction techniques, such as pulsed electric field extraction [55], ultrasound-assisted extraction [29,37,44], pressurized liquid extraction [29] and high-pressure carbon dioxide extraction [58], present higher extraction yields than the conventional solvent extraction. In several previous studies, researchers have also compared the yield of anthocyanins extracted from different cultivars of PSP, demonstrating that the total anthocyanin contents in different cultivars of PSP are very different [25,27,60,61,62,63,64]. In some studies, researchers have compared the yield of anthocyanins extracted from different parts (i.e., root and leaf) of PSP, revealing that the root has a higher total anthocyanin content than the leaf [63,65]. Interestingly, the outer and inner layers of PSP root also have different total anthocyanin contents [64]. It is worth noting that the root of PSP can be pre-cooked by different means, such as baking, boiling, steaming, frying and pressure cooking, before the extraction of PSPAs [24,25,26,27,28]. Results show that the total anthocyanin content of PSP is remarkably affected by the pre-cooking method and precooking condition of PSP. This is because pre-cooking treatment can soften the tissue and inactive polyphenol oxidase in PSP, which can facilitate the extraction and stabilization of PSPAs. However, pre-cooking treatment has a thermal degradation effect on PSPAs. Therefore, the pre-cooking condition of PSP should be accurately controlled. To sum up, the extraction yield of PSPAs is influenced by the extraction method, extraction condition, cultivar, part and even pre-cooking treatment of PSP.

## 3. Isolation, Characterization and Purification of PSPAs

### 3.1. Isolation of PSPAs

The obtained anthocyanin-rich PSP extract contains several unwanted substances, such as sugar, organic acid, protein, mineral and other polyphenols. So PSPAs should be isolated from the extract to obtain the mixture of anthocyanin monomers. To date, PSPAs have been isolated from the extract by different means, such as the aqueous two-phase system (ATPS), membrane separation and column chromatography.

#### 3.1.1. ATPS

**Figure 2:**
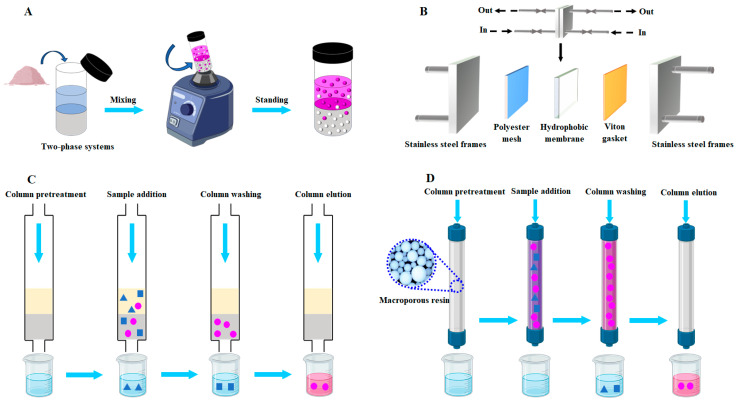
Different methods used for the isolation of PSPAs: ATPS (**A**), membrane separation (**B**), C18 solid phase extraction cartridge (**C**) and macroporous adsorption resin (**D**).

ATPS, a unique liquid–liquid extraction technique, has been widely used in the extraction and isolation of anthocyanins [66]. The advantages of ATPS include low cost, a simple operation process and high selectivity. As displayed in Figure 2A, ATPS consists of two immiscible phases that are formed by mixing two aqueous solutes beyond their critical concentrations. The typical ATPS used for the extraction and isolation of PSPAs is composed of ammonium sulphate aqueous solution and ethanol aqueous solution [32,51]. These two aqueous solutions are intensively mixed and allowed to stand still with the formation of two separated phases, consisting of a bottom salt-rich phase and a top ethanol-rich phase. Since different substances in the extract have distinct partition coefficients in two aqueous phases, PSPAs can simply be isolated from other substances in the extract. Considering PSPAs have a high partition coefficient in the ethanol aqueous solution, the anthocyanin-rich top phase is recovered for further analysis.

#### 3.1.2. Membrane Separation

Membrane separation technique, due to its low cost, easy operation, nondestructive nature and high productivity, has received increasing attention in the isolation of anthocyanins [67]. The conventionally used pressure-driven membrane separation technique includes micro-filtration, ultra-filtration and nanofiltration, and is based on the different molecular or particle sizes of PSPAs and impurities (Figure 2B). The pore sizes of micro-filtration, ultra-filtration and nano-filtration membranes are 100–10,000 nm, 2–100 nm and 0.5–2 nm, respectively [68]. Accordingly, the micro-filtration membrane is capable of removing the suspended particles with sizes larger than 100 nm. The ultra-filtration membrane can separate substances with a molecular weight between 1000 and 20,000 Da. The nano-filtration membrane is often used to recover substances (e.g., anthocyanins) with molecular wights lower than 1000 Da [68]. However, only one study has reported the isolation of PSPAs by using the membrane separation technique [69]. PSP extract was applied to a micro-filtration membrane with a pore size of 100 nm and the obtained permeate was further applied to the ultra-filtration membrane with a molecular weight cut-off of 10,000 Da. The resultant permeate was finally applied to a nano-filtration membrane with a molecular weight cut-off of 400–600 Da [69]. Results show that the total anthocyanin content in different membrane filtration permeates gradually decreased, with ultra-filtration and nano-filtration membranes having relatively higher isolation efficiency.

#### 3.1.3. Column Chromatography

The principle of column chromatography is based on the fact that PSPAs and impurities have distinct distribution coefficients in solid and mobile phases [70]. The commonly used column fillers include C18 solid phase extraction cartridge (Figure 2C) and macroporous adsorption resin (Figure 2D). The commercially available Sep-Pak solid C18 solid phase extraction cartridge is often selected for the isolation of PSPAs [24,26,28,29,58,62,64]. The cartridge is activated with methanol and acidified water. After the sample is loaded into the cartridge, the cartridge is first eluted with acidified water and then eluted with ethyl acetate to remove non-anthocyanin polyphenolic substances and other polar compounds. Finally, the cartridge is eluted with acidified methanol to recover PSPAs. The residual methanol in the eluate can be simply removed by rotary evaporation. Different kinds of macroporous adsorption resins, such as AB-8 resin [61,71,72,73], Amberlite XAD-7 resin [31,74], X-5 resin [75], Diaion HP-20 resin [76], HP2MGL resin [77] and D101/AB-8 composite resins [47] have been used in the isolation of PSPAs, which is because macroporous resins have the advantages of low cost, large adsorption capacity and reusability. In general, macroporous resins are first eluted with water to remove sugars, organic acids, proteins and minerals, and then eluted with acidified aqueous ethanol solutions. In order to achieve better isolation outcomes, two different types of columns can be combined for the isolation of PSPAs. For example, Luo et al. [71] and Zhang et al. [72] first isolated PSPAs by an AB-8 macroporous resin column and the obtained eluate was further loaded onto a Daisogel ODS C18 packing column, which was sequentially eluted with water and different concentrations of acidified methanol aqueous solutions (20%, 30%, 40% and 50%, *v*/*v*). Similarly, Terahara et al. [76] sequentially isolated PSPAs on a Diaion HP-20 macroporous resin column and Sephadex LH-20 gel column.

### 3.2. Characterization of PSPAs

The isolated PSPAs are a mixture of anthocyanin monomers, which are normally qualitatively and quantitatively analyzed by high-performance liquid chromatography-mass spectrometry (HPLC-MS) and HPLC-UV detector, respectively. In the HPLC system, the reversed-phase C18 chromatographic column is often used for the separation of individual anthocyanin monomers and the column is gradiently eluted with a mixture of acidified water and acetonitrile. For the qualitative identification of PSPAs, different MS techniques including electrospray ionization mass spectrometry (ESI-MS), electrospray ionization-quadrupole-time-of-flight mass spectrometry (ESI-QTOF-MS) and MS/MS are often applied. For the quantitative analysis of PSPAs, UV detectors, including photo-diode array (PDA) and diode array detector (DAD), are normally selected with the detection wavelength setting at 530 nm.

**Figure 3:**
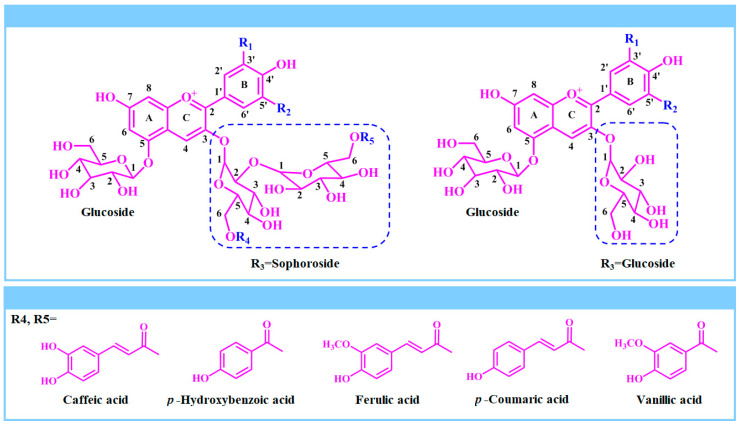
The skeleton of PSPAs and their glycosylated and acylated substituents.

The composition of PSPAs has been characterized by different researchers [24,25,26,27,28,29,31,32,35,48,50,58,60,61,62,63,64,65,71,74,75] and is summarized in Table S1. In total, over 60 kinds of PSPAs have been identified up to now. In general, PSPAs are the glycosylated and acylated products of anthocyanidins, such as cyanidin, peonidin, pelargonidin and delphinidin. Among different anthocyanidins, cyanidin and peonidin are commonly found in PSPAs. As displayed in Figure 3, 3-sophoroside-5-glucoside is the common glycosylation form of PSPAs. By contrast, the acylation form of PSPAs is more diverse, including monoacylation and diacylation with ferulic, caffeic, *p*-hydroxybenzoic, *p*-coumaric and vanillic acids [78]. It is worth noting that most anthocyanins in PSP are acylated ones. Thus, PSPAs are considered as highly acylated anthocyanins.

Notably, the composition of PSPAs is influenced by different factors, including the extraction method, cultivar, part and pre-cooking of PSP (Table S1). For instance, Cai et al. [29] identified twelve anthocyanins from the root of PSP, and found that PSPAs obtained by pressurized liquid extraction had a higher content of diacyl anthocyanins than PSPAs obtained by conventional solvent extraction and ultrasound-assisted extraction. Similarly, Lao et al. [58] found that PSPAs obtained by high-pressure carbon dioxide extraction had different compositions than PSPAs obtained by conventional water or aqueous ethanol extraction. These two studies reveal the influence of extraction method in the composition of PSPAs. The impact of PSP cultivar on the composition of PSPAs has been deeply investigated by several researchers, demonstrating that PSPAs extracted from different cultivars of PSP have different compositions [25,27,60,61,62,63,74]. These studies have mainly focused on PSPAs extracted from the root [25,27,60,61,62,64,74] and minor attention has been paid to PSPAs extracted from the leaf [63,65]. Several studies have reported that the composition of PSPAs in the leaf and root of PSP is different [31,63,65]. Interestingly, Im et al. [64] documented that PSPAs in the inner and outer layers of the root were different. These three studies reveal different parts of PSP had distinct compositions of PSPAs. It is worth noting that the pre-cooking of PSP has a big impact on the composition of PSPAs [24,25,26]. Two representative studies were performed by Kim et al. [26] and Xu et al. [28], demonstrating that the composition of PSPAs greatly changed after PSP was steamed, roasted and pressure cooked.

### 3.3. Purification of PSPAs

**Figure 4:**
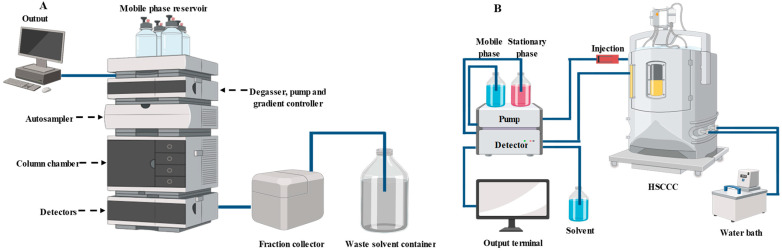
The purification of PSPAs by semi-preparative HPLC (**A**) and high-speed countercurrent chromatography (**B**).

Although many studies have reported the composition of PSPAs, only a few studies have focused on the large-scale preparation (i.e., purification) of individual anthocyanin monomers [71,72,73,74,75,77,79]. Nowadays, the individual anthocyanins are hardly available and are expensive, which is because the purification of anthocyanins is a big challenge [80]. It has been demonstrated that semi-preparative HPLC and high-speed countercurrent chromatography (HSCCC) are useful for the purification of PSPAs. Similarly to analytic HPLC, semi-preparative HPLC utilizes a reversed-phase C18 chromatographic column to isolate the individual anthocyanin monomers under the same mobile phase gradient and detection conditions (Figure 4A). However, the column size, sample injection volume and flow rate of semi-preparative HPLC are much higher than those of analytic HPLC [71,72,73,75]. As for HSCCC, it is a support-free liquid–liquid partition chromatographic technique for purifying PSPAs [70]. The two-phase solvent systems including tert-butyl methyl ether/n-butanol/acetonitrile/water/trifluoroacetic acid [74,79] and n-butanol/ethyl acetate/water/acetic acid [77] are commonly used for the purification of PSPAs. Prior to purification, the solvent system is equilibrated in a separatory funnel with the formation of the upper and the lower phases. The obtained upper and lower phases are separated and used as the stationary phase and the mobile phase, respectively (Figure 4B). HSCCC is different from semi-preparative HPLC in that it avoids the irreversible adsorption of samples on the solid phase support and is suitable for the fast and large-scale preparation of PSPAs [74,77,79]. It should be noted that only the main components of PSPA monomers have been purified by semi-preparative HPLC and HSCCC in existing studies. Moreover, the detailed molecular structures of the obtained PSPA monomers are often further characterized by HPLC-MS and nuclear magnetic resonance (NMR).

## 4. Functional Properties of PSPAs

### 4.1. Antioxidant and Antimicrobial Activity

PSPAs, belonging to polyphenols, are potent antioxidant agents. Until now, several researchers have evaluated the antioxidant activity of anthocyanin-rich PSPA extract by using different in vitro methods [29,34,61,64], such as 2,2-diphenyl-1-picrylhydrazyl (DPPH) radical scavenging activity, 2,2′-azino-bis(3-ethylbenzothiazoline-6-sulfonate) (ABTS) radical scavenging activity, hydroxyl radical scavenging activity, reducing power, linoleic acid autoxidation inhibition activity, lipid peroxidation inhibition activity, ferric reducing antioxidant capacity, oxygen radical absorbance capacity, etc. Existing studies have revealed that the antioxidant activity of anthocyanin-rich PSPA extract is influenced by the cultivar and part of PSP [61,64] as well as the extraction method and extraction solvent of PSP [29]. It should be noted that the antioxidant activity of anthocyanin-rich PSP extract is attributed to not only anthocyanins but also other types of polyphenols. For example, Zhao et al. [73] purified five colorless caffeoyl compounds from PSP by semi-preparative HPLC and found that the compounds had strong antioxidant activity. Results showed di-caffeoylquinic acid had stronger antioxidant activity than mono-caffeoylquinic acid. Therefore, the isolation and purification of PSPAs are required to obtain anthocyanin monomers before analyzing the antioxidant activity of PSPAs. However, only a few studies have investigated the antioxidant activity of the purified PSPA monomers [71,75]. Luo et al. [71] purified four major PSPA monomers by using semi-preparative HPLC and further compared the antioxidant activity of these monomers, which decreased in the order of peonidin-3-dicaffeoyl-sophoroside-5-glucoside > cyanidin-3-caffeoyl-feruloyl sophoroside-5-glucoside > peonidin-3-caffeoyl-feruloyl-sophoroside-5-glucoside > peonidin-3-caffeoyl-*p*-hydroxybenzoyl-sophoroside-5-glucoside. This study reveals that the mother nucleus of PSPAs affects their antioxidant activity, with cyanidin showing higher antioxidant activity than peonidin. Moreover, different acylated residues strengthen the antioxidant activity of the mother nucleus at different degrees, which follow the order of caffeoyl > feruloyl > *p*-hydroxybenzoyl. Sun et al. [75] compared the antioxidant activity of five major peonidin-based PSPA monomers through four antioxidant assays. They found the antioxidant activity of PSPA monomers decreased in the order of peonidin-3-caffeoyl-*p*-hydroxybenzoyl-sophoroside-5-glucoside > peonidin-3-caffeoyl-feruloyl-sophoroside-5-glucoside > peonidin-3-caffeoyl-sophoroside-5-glucoside > peonidin-3-feruloyl-sophoroside-5-glucoside > peonidin-3-sophoroside-5-glucoside. This study further reveals that di-acylated PSPAs have higher antioxidant activity than mono-acylated PSPAs and non-acylated PSPAs. As compared with the antioxidant activity of PSPAs, the antimicrobial activity of PSPAs has been less investigated [81,82]. Meanwhile, studies have only focused on the antimicrobial activity of anthocyanin-rich PSPA extract. Existing studies have demonstrated the antimicrobial activity of anthocyanin-rich PSPA extract is related to the cultivar of PSP [81]. Nonetheless, the antimicrobial activity of the purified PSPA monomers has not been investigated yet.

### 4.2. Color-Changing Ability

**Figure 5:**
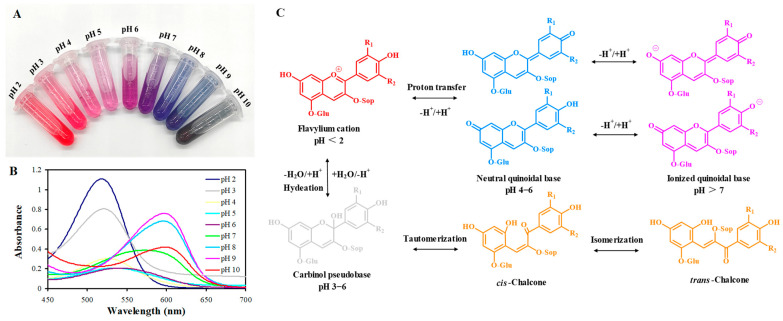
The color change (**A**) and visible spectra (**B**) of PSPAs from pH two to ten, and the pH-dependent structural transformation of cyandin-3-sophoroside-5-glucoside at different pH values (**C**).

The color-changing ability of PSPAs has received increasing attention in recent years, because the color-changing ability is vital for PSPAs in intelligent packaging. Since PSPAs are highly acylated anthocyanins, they have superior color-changing ability over the non-acylated anthocyanins [17]. The color-changing ability of PSPAs is often tested in different pH solutions, where PSPAs show a trend of red → pink → purple → blue → green from two to ten (Figure 5A). The color changes of PSPAs can be detected by measuring the visible spectrum of PSPA solutions. As displayed in Figure 5B, when pH increases from two to six, the characteristic absorption peak of PSPAs at 530 gradually decreases. When pH further increases from seven to ten, a new absorption peak of PSPAs appears at 605 nm and gradually increases [22,78,83]. The observed red-shift phenomenon with increasing pH can be explained by the different structural forms of PSPAs at different pHs, such as flavylium cations, carbinol pseudobases and neutral quinone bases in acidic conditions and anionic quinone bases and cis-/trans-chalcone in alkaline conditions [78]. These structural forms of PSPAs can transform when the pH changes, which is accompanied by the color changes of PSPAs (Figure 5C).

The color-changing ability of PSPAs is altered by adding other colorants, such as curcumin [84] and betacyanins [85]. Since anthocyanins have superior color-changing ability over other types of colorants, the mix of PSPAs with other colorants normally results in worse color-changing performances [84,85]. The biogenic silver nanoparticles are reported to have a negative impact on the color-changing ability of PSPAs, making the PSPA solutions become yellow at different pHs [86]. A similar phenomenon is observed when commercial zinc oxide nanoparticles are added into PSPAs [87]. In contrast, tannic acid can strengthen the color-changing ability of PSPAs, which is based on the intermolecular pigmentation between tannic acid and PSPAs through π-π stacking [88]. For the first time, Li et al. [89] demonstrated that the color-changing ability of PSPAs was altered after PSP was subjected to different pre-cooking treatments. They found that PSPAs in the steamed PSP maintained color-changing ability while PSPAs in the boiled and roasted PSP showed a decline in color-changing ability, which was because different pre-cooking treatments had different impacts on the stability of PSPAs [89].

## 5. Biopolymer-Based Smart Packaging Films Containing PSPAs

### 5.1. Preparation Methods

As summarized in Table 2, natural biopolymers derived from plants (e.g., starch, pectin, cellulose derivatives, zein, agarose, carrageenan, locust bean gum and alginate), animals (e.g., chitosan and gelatin) and microorganisms (e.g., gellan gum, pullulan and riclin) have been used as the solid supports of PSPAs to fabricate smart packaging films. Most of these biopolymers are hydrophilic and can be dissolved in water. In order to improve the hydrophobicity of the hydrophilic biopolymer-based films, polyvinyl alcohol (PVA) is often blended with natural biopolymers to yield a binary film matrix. Hydrophobic biopolymers, such as cellulose derivatives (ethyl cellulose and cellulose acetate), are occasionally selected as a film matrix to fabricate water-proof films, where these biopolymers are dissolved in anhydrous ethanol or acetone [47,90,91].

In most cases, the anthocyanin-rich PSP extract is used for smart packaging film production. However, the anthocyanin composition of PSP extract is seldom analyzed by researchers [17,23,92]. Notably, no study has reported the production of smart packaging films using the purified PSPA monomers up to now. Apart from anthocyanin-rich PSP extract, the dried PSP powder without anthocyanin extraction is another choice to fabricate smart packaging films [23,93]. In some circumstances, PSP is first subjected to pre-cooking treatments and then milled into powder, and the cooked PSP powder is further used for smart packaging film production [89,94,95]. It is worth noting that the pre-cooking treatments are beneficial for the gelatinization of starch and the inactivation of polyphenol oxidase in PSP, which are useful to increase the stability of PSPAs. Meanwhile, the preparation of smart packaging films based on the cooked PSP powder avoids the tedious PSPA extraction processes and thus saves remarkably on the production cost [89]. Despite this, the films produced from the cooked PSP powder have low homogeneity and mechanical strength, which are due to the presence of crude fibers in the cooked PSP powder [95].

The film-forming solutions are generated by mixing anthocyanin-rich PSP extract or PSP powder with biopolymer-based solutions. Cross-linking agents, such as sodium trimetaphosphate, CaCl_2_ and oxidized alginate, are sometimes added into the film-forming solutions to increase the stability of the films through different action mechanisms [96,97,98,99,100]. For example, the phosphate groups in sodium trimetaphosphate can react with the hydroxyl groups of biopolymers through phosphodiester bonds [97]. The divalent cation (Ca^2+^) of CaCl_2_ can react with the negatively charged biopolymers (e.g., sodium alginate) [98,99,100]. The aldehyde groups in oxidized alginate can react with the amino groups of biopolymers (e.g., chitosan and gelatin) through Schiff-base linkages [96]. The cross-linking can greatly shorten the distance between biopolymer chains, resulting in a dense gel network. It is worth noting that these chemical cross-linking agents are more or less toxic and have the risk of migrating into the food, especially when the prepared packaging films come into contact with the food. In this regard, safer cross-linking methods, such as physical and enzymatic cross-linkings, could be utilized in future studies.

As listed in Table 2, different kinds of reinforcing agents including essential oils, polyphenols, nanomaterials and other types of colorants are added to elevate the function of the films in different aspects. Only a few researchers have adjusted the pH of the film-forming solutions [23,78,83,84,99,101,102,103]. However, the impact of the pH of the film-forming solutions on the performance of the films has not been investigated. The rheological properties (e.g., elastic modulus and loss modulus) of the film-forming solutions are sometimes determined, which is helpful for understanding the interactions between biopolymers and PSPAs [23,83,104]. Notably, PSPAs can interact with biopolymers by different means, depending on the charged character of biopolymers. For instance, PSPAs normally interact with the uncharged biopolymers (e.g., starch, locust bean gum, agarose and pullulan) through hydrogen bonds. In addition, PSPAs usually interact with the positively charged biopolymers (e.g., chitosan) and the negatively charged biopolymers (e.g., pectin, carrageenan, alginate and gellan gum) through hydrogen bonds as well as electrostatic interactions. The additional plasticizer and reinforcing agents (e., essential oils, polyphenols and nanomaterials) can also interact with PSPAs and biopolymers through hydrogen bonds and/or electrostatic interactions.

**Figure 6:**
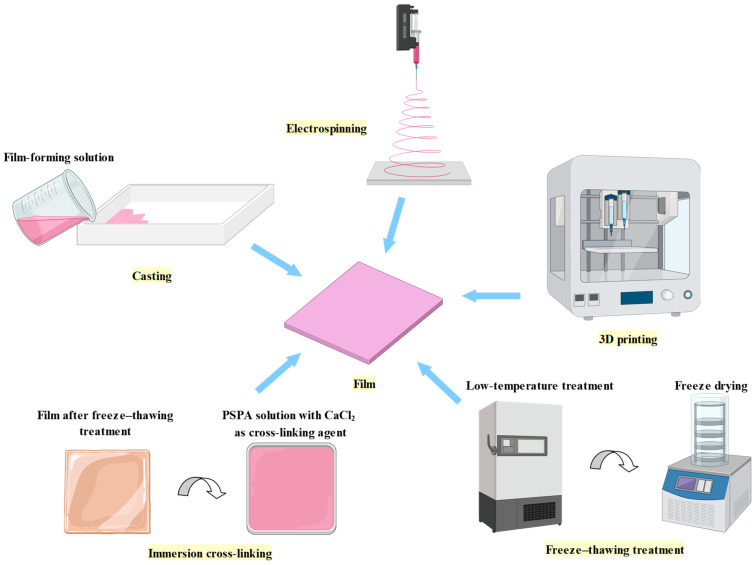
Different means to prepare biopolymer-based smart packaging films containing PSPAs.

As summarized in Table 2 and shown in Figure 6, solvent casting is adopted by most researchers to fabricate biopolymer-based smart packaging films containing PSPAs, where the prepared film-forming solutions are normally cast and dried in petri dishes. This is because the solvent casting method is simple and does not require expensive instruments. Bi-layer films consisting of a PSPA-incorporated indicator layer and an antibacterial layer can be developed by layer-by-layer casting technology [88,103,105]. The bi-layer films strengthen the antibacterial activity and mechanical strength of the mono-layer films. Except for solvent casting, some novel methods have also been used to produce biopolymer-based smart packaging films containing PSPAs. For instance, the electrospinning technique is used to develop smart packaging films with PSPAs being encapsulated in nanofibers [91,105,106]. Three-dimensional printing is a feasible method for designing personalized smart packaging apparatus based on biopolymers and PSPAs [83]. However, both electrospinning and 3D printing require specific and expensive instruments and thus are seldom adopted by most researchers. Recently, smart packaging materials containing PSPAs have been produced by freeze-drying treatment. For example, the film-forming solutions can be subjected to three freeze–thawing treatments and one freeze-drying treatment to prepare absorbent pads for preserving meat products [86,104]. The freeze–thawing treated hydrogels can be also immersed in PSPA solution, where PSPAs are adsorbed into the hydrogels [99].

### 5.2. Physical Properties

The physical properties of the films containing PSPAs greatly affect their applicability. As summarized in Table 2, the incorporation of PSPAs can alter the physical properties of biopolymer-based films to different degrees. However, the results are inconsistent and even contradictory, which is because the physical properties of the films are affected by many factors. Several studies have demonstrated the physical properties of the films are greatly affected by the amount of PSPAs in the films [23,97,107,108]. Except for PSPAs, the film matrix is a nonnegligible factor affecting the physical properties of the films. For instance, Yong et al. [109] compared the physical properties of locust bean gum-, chitosan- and carrageenan-based films containing PSPAs. They found the type of film matrix greatly influenced the color, barrier and mechanical properties of the films [109]. Similarly, Sohany et al. [107] compared the physical properties of starch/PSPA films and starch/PSP peel powder/PSPA films. Results showed that the starch/PSP peel powder/PSPA films had a darker color and higher swelling degree and water vapor permeability, but lower tensile strength and elongation at break than starch/PSPA films. Recently, Zong et al. [110] revealed that the ratio of starch and gelatin had a big impact on the physical properties of starch/gelatin/PSPA films, with the starch/gelatin ratio of 1:1 film showing the lowest water vapor permeability but the highest elongation at break. Likewise, Ke et al. [111] found that the physical properties of polyvinyl alcohol/sodium alginate/carboxymethyl cellulose/PSPA films were affected by the ratio of polyvinyl alcohol and sodium alginate/carboxymethyl cellulose blend, with the polyvinyl alcohol and sodium alginate/carboxymethyl cellulose ratio of 6:4 film exhibiting the lowest moisture content, water vapor permeability and elongation at break but the highest water contact angle. Wu et al. [106] reported that, with an increasing gelatin/ethyl cellulose ratio, the water contact angle deceased but the swelling ratio, water solubility and water vapor permeability of ethyl cellulose/gelatin/PSPA nanofiber films increased. The above evidence shows that the physical properties of the films are influenced by the composition of the film matrix.

The physical properties of the films containing PSPAs can also be altered by adding reinforcing agents. The direct addition of essential oils can greatly decrease the hydrophilicity, light transmittance and tensile strength of the films [94,112]. When essential oils are encapsulated in Pickering emulsions, they increase the water contact angle and elongation at break of the films [88]. Castor oil, one kind of vegetable oil, is able to increase the tensile strength and hydrophilicity of ethyl cellulose/PSPA films [47]. Dong et al. [113] revealed that the incorporation of quercetin-loaded chitosan nanoparticles into agar/sodium alginate/PSPA films decreased the water solubility, water vapor permeability, oxygen permeability and light transmittance of the films. He et al. [86] found that biogenic silver nanoparticles increased the water solubility and water contact angle of polyvinyl alcohol/paper fiber/PSPA pads. Pang et al. [114] reported ε-polylysine decreased the moisture content, light transmittance and tensile strength but increased the water vapor permeability of chitosan/hydroxypropyl methyl cellulose/PSPA films. Recently, Ding et al. [96] found that the addition of oxidized alginate into gelatin/PSPA films increased the tensile strength but decreased the swelling ratio, water vapor permeability and elongation at break of the films, which was attributed to the cross-linking effect of oxidized alginate. Pang et al. [90] documented that eugenol increased the water contact angle and elongation at break but decreased the water vapor permeability, oxygen permeability and tensile strength of cellulose acetate films supplemented with a PSPA-loaded γ-cyclodextrin metal-organic framework. Gao et al. [85] reported the addition of dragon fruit betacyanins increased the water vapor barrier and mechanical properties of carrageenan/PSPA films. Meanwhile, the physical properties of the carrageenan/PSPA/betacyanin films were related to the ratio of PSPAs and betacyanins in the films. In a recent study, Li et al. [98] revealed that the color, moisture content, water solubility, water vapor permeability, light transmittance, tensile strength and elongation at break were influenced by the drying temperature of sodium alginate/PSPA films. This study indicates that the film preparation conditions have some impacts on the physical properties of the films.

### 5.3. Antioxidant and Antibacterial Activity

To assess the active packaging potential of the films containing PSPAs, the antioxidant activity of the films is often tested by DPPH and/or ABTS radical scavenging activity while the antibacterial activity of the films is normally evaluated using the inhibition rate method or agar diffusion plate method (Table 2). Since PSPAs themselves are potent antioxidant agents, the films containing PSPAs often present good antioxidant activity. Meanwhile, the antioxidant activity of the films is positively correlated to the amount of PSPAs in the films [103,108]. The antioxidant activity of the films can be elevated by incorporating additional bioactive compounds, such as carvacrol [105], betacyanins [85] and eugenol [90]. Interestingly, some polysaccharides, such as riclin, an expolysaccharide produced by Agrobacterium, can act as not only a film matrix but also as an antioxidant agent [102]. As for the films prepared from the cooked PSP powder, their antioxidant activity is associated with the pre-cooking method [89] and the cultivar of PSP [95].

Considering that the antibacterial activity of the films containing PSPAs is very low, several researchers have managed to enhance the antibacterial activity of the films by adding extra antimicrobial agents, such as essential oils [94,112], carvacrol [105], quercetin-loaded chitosan nanoparticles [113], biogenic silver nanoparticles [86], zinc oxide nanoparticles [87,92], ε-polylysine [106,114] and magnolol [103]. The direct addition of essential oils remarkably elevates the antibacterial activity of the films against *E. coli* and *L. monocytogenes* [94,112]. The antibacterial activity of the films containing PSPAs can also be elevated by covering an antibacterial layer through a layer-by-layer casting technique [103,105]. Interestingly, the addition of polyphenols or polyphenol-loaded nanoparticles can elevate both the antibacterial and antioxidant activity of the films [103,105,113].

Notably, the antioxidant and antibacterial activity of the films depend on the release of PSPAs from the films. In most cases, the release of PSPAs from the films is tested in different kinds of food simulants, including distilled water (high water content food), 3% acetic acid (acidic food), 50% ethanol (lower fatty food) and 95% ethanol (fatty food). Guo et al. [105] measured the release profile of PSPAs from pullulan-based films in 85% ethanol aqueous solution, and found that the cumulative release ratio of PSPAs increased with an increasing PSPA amount in the films. Meanwhile, the release mechanism of PSPAs from the films followed Fickian diffusion law [105]. Sohany et al. [107] reported that PSPAs were more rapidly released into the aqueous solutions (e.g., distilled water and 3% acetic acid) than into the ethanol solutions, which was because PSPAs and the film matrix (i.e., PSP starch) were both hydrophilic. This study suggests that the release of PSPAs is influenced by the type of food simulants. In some circumstances, the release of PSPAs from the films is tested in the simulated gastrointestinal fluids with pHs of 2.0, 6.0 and 7.4 [23]. The researchers found that the films released PSPAs more slowly in pH 2.0 fluid than in pH 6.0 and 7.4 fluids, which could be explained by the better swelling ability of the films at higher pHs. This study reveals the release of PSPAs is affected by the pH of the simulants.

### 5.4. pH Sensitivity

Since PSPAs have color-changing ability, the pH sensitivity of the films containing PSPAs is a big concern of the researchers. In most studies, the pH sensitivity of the films is tested by dipping the films in buffer solutions or exposing the films to acid–base gases. Obvious color changes can be observed when the external acid–base condition changes. Except for color changes, visible spectral changes are normally noted for the films containing PSPAs, where the spectra of the films shift from 530 nm to 605 nm after the films are exposed to alkaline conditions [22,78]. Interestingly, Choi et al. [22] found that the absorbance ratio of agar/starch/PSPA films at 605 and 530 nm had an exponential relationship with the pH of buffer solutions. Similarly, Huang et al. [101] found that the absorbance ratio of agarose/PSPA films at 605 and 535 nm had a typical time-dependent sigmoid trend. Therefore, the response ability of the films to pH changes can be effectively evaluated. Huang et al. [101] demonstrated that the response ability of agarose/PSPA films to volatile ammonia was influenced by the amount of PSPA in the films, the pH of film-forming solutions, the concentration of ammonia solution, environmental humidity and the temperature of the testing environment. Some researchers even tested the pH sensitivity of the films by hanging the films above the nutrient agar plate inoculated with *E*. *coil* [23]. The researchers found that the pH of the bacterial culture medium gradually increased because the beef extract and peptone in the culture medium were utilized by bacteria to produce amines. As a result, the films changed from purple to gray-green after the bacteria were incubated for 24 h [23]. The colorimetric response of the films is useful for detecting bacterial growth in meat products, and thus for indirectly predicting the freshness of foods. Recently, Yan et al. [115] found corn starch/carboxymethyl cellulose/PSPA films were sensitive to different concentrations of hydrogen peroxide. The films changed from red to pink and yellow with an increasing concentration of hydrogen peroxide, which was helpful for indicating the oxidation degree of edible oils.

Existing studies have demonstrated that the pH sensitivity of the films is greatly affected by the amount of PSPAs in the films, with the films containing high contents of PSPAs displaying deeper chrominance [100,102,104,107,108,116]. Interestingly, Li et al. [89] reported that the pH sensitivity of the cooked PSP powder-based films was influenced by the pre-cooking method of PSP, with the steamed and boiled PSP powder-based films showing better pH sensitivity than the roasted PSP powder-based film. Recently, different steamed PSP powder-based films were prepared by Yun et al. [95] from nine cultivars of PSP. The researchers found that the pH sensitivity of the films was associated with the cultivar of PSP, which was because different cultivars of PSP had different total anthocyanin contents and anthocyanin compositions. The film matrix is another important factor affecting the pH sensitivity of the films containing PSPAs. For instance, Sohany et al. [107] found the color changes of PSPAs immobilized in a starch/PSP peel powder blend matrix were more profound than those of PSPAs immobilized in a starch matrix. Similarly, Yong et al. [109] revealed that PSPAs embedded in locust bean gum- and carrageenan-based film matrices showed better pH sensitivity than PSPAs embedded in a chitosan-based film matrix. However, when PSPAs are immobilized in hydrophobic polymer-based films, the films do not show obvious color changes because the hydrophobic matrix restricts the contact of PSPAs with hydrogen ions and hydroxide ions [47].

In addition to PSPAs and the film matrix, reinforcing agents have big impacts on the pH sensitivity of the films. For example, the addition of biogenic silver nanoparticles made polyvinyl alcohol/paper fiber/PSPA pads tend to present as a brownish-yellow color in different buffer solutions [86]. However, in other studies, researchers found that the pH sensitivity of the films was not significantly changed by essential oils, castor oil, eugenol and modified aramid nanofibers [47,94,99,112]. Recently, Wen et al. [91] and Wu et al. [106] revealed that the pH sensitivity of the films was influenced by the film preparation method, with the electrospinning nanofiber film showing more obvious ammonia sensitivity than the casting film. This was because the electrospinning nanofiber film had a larger specific surface area and higher porosity in comparison with the casting film. The film preparation conditions have a big impact on the pH sensitivity of the films. For example, Li et al. [98] found the sodium alginate/PSPA films dried at low temperatures (25–35 °C) showed more obvious color changes than the films dried at high temperatures (40–55 °C), which was due to the destructive effect of high temperatures on PSPAs. Recently, Li et al. [88] documented that the bi-layer films containing PSPAs showed different color-changing profiles when the films are exposed to ammonia, dimethylamine and trimethylamine, respectively. This study demonstrates that the pH sensitivity of the films is affected by the testing conditions of the films.

Notably, the pH sensitivity of the films can be altered by adding other colorants, such as curcumin [84] and betacyanins [85]. Chen et al. [84] found that starch/polyvinyl alcohol/PSPA films became less sensitive to ammonia vapor after curcumin was added into the films. The same researchers also demonstrated that the ammonia sensitivity of starch/polyvinyl alcohol/PSPA films was enhanced by adding glycerol, which was because glycerol acted as a hydrophilic plasticizer that could adsorb moisture and allow ammonia vapor to enter the films more easily [84]. In a similar study, Gao et al. [85] documented that the pH sensitivity of carrageenan/PSPA films decreased after dragon fruit betacyanins were added into the films. Recently, Pang et al. [90] demonstrated that the pH sensitivity of cellulose acetate/PSPA films declined after PSPAs were encapsulated in a γ-cyclodextrin metal-organic framework, which was because the wall material had a shielding effect on the color of PSPAs. This study reveals that the pH sensitivity of the films is affected by the encapsulation of PSPAs.

In some studies, researchers have investigated the reusability of the films by exposing the films in turn to acetic acid and ammonia vapor for several rounds [90,92,96,105,113]. The films that can maintain good pH sensitivity after several rounds of acetic acid and ammonia vapor treatments are considered as reusable.

### 5.5. Color Stability

Since PSPAs are unstable substances, the color stability of the films containing PSPAs should be measured. The stability of the films is often tested by storing the films with controlled temperature, humidity and illumination conditions [84,85,88,97,102,113]. During the storage period, the total color difference (Δ*E*) and relative color change (S) are often recorded based on CIE-Lab and RGB color coordinates. Jiang et al. [97] investigated the impacts of storage temperature (−20 and 25 °C), humidity (43% and 83%) and illumination (with and without lighting) on the stability of carboxymethyl cellulose/starch/PSPAs films, and found that the stability of the films was more significantly affected by storage temperature. Similarly, Capello et al. [117] reported no obvious color changes were noted for chitosan/polyvinyl alcohol/PSPA films after photodegradation at 20 °C for 8 days.

Notably, the film matrix is an important factor affecting the color stability of the films containing PSPAs. Yong et al. [109] demonstrated that the PSPAs embedded in a locust bean gum-based film matrix had higher stability than PSPAs embedded in chitosan- and carrageenan-based film matrices. Different types of reinforcing agents have different impacts on the stability of the films containing PSPAs. Chen et al. [84] compared the stability of starch/polyvinyl alcohol films containing curcumin and/or PSPAs after storage in darkness at 25 °C for 6 months. They found that the films supplemented with curcumin and PSPAs showed higher stability than the films supplemented with PSPAs alone, which was because curcumin was more stable than PSPAs. This study indicates the stability of the films containing PSPAs can be improved by adding curcumin. Likewise, Gao et al. [85] demonstrated that the stability of the films containing PSPAs was slightly elevated by adding betacyanins. However, Wang et al. [47] found that the stability of ethyl cellulose/PSPA films was little influenced by castor oil, where castor oil acted as a plasticizer in the films.

In recent years, increasing attention has been paid to the stability enhancement of the films containing PSPAs. Since PSPAs are water soluble substances, they can easily leach from the films in highly humid packaging environments. Up until now, only a few studies have managed to inhibit the leaching of PSPAs from the films. Wang et al. [47] immobilized PSPAs in an ethyl cellulose-based matrix and found that the leaching of PSPAs from the films into water was retarded, which was ascribed to the hydrophobic nature of the ethyl cellulose matrix. In another study, Zhai et al. [83] first dissolved PSPAs in agar-based hydrogel and then encapsulated the hydrogel in beeswax and glyceride monooleate-based oleogel, and the resultant bigel showed the anti-leaching property of PSPAs in water. Recently, Ding et al. [96] found that the leaching of PSPAs from gelatin-based films was effectively retarded by adding oxidized alginate, which was because oxidized alginate formed Schiff-base linkage and hydrogen bonds with gelatin. In the study of Pang et al. [90], the researchers managed to elevate the stability of PSPAs by encapsulating PSPAs in a γ-cyclodextrin metal-organic framework, which was ascribed to the protective effect of wall material on PSPAs. The above findings suggest that the leaching of PSPAs from the films can be inhibited by hydrophobic treatment on the films, the cross-linkage of the film matrix and the encapsulation of PSPAs.

### 5.6. Applications

**Figure 7:**
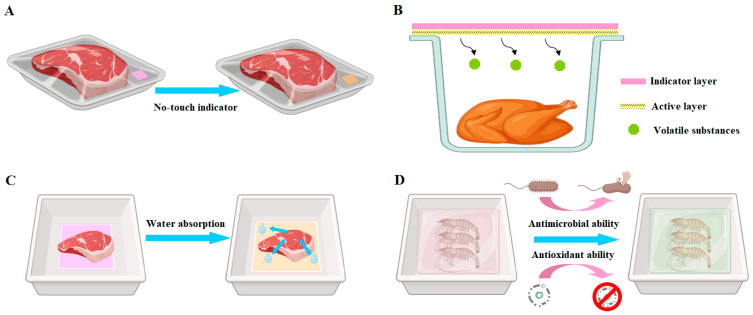
Different application forms of biopolymer-based smart packaging films containing PSPAs: no-touch indicator (**A**), indicator supplemented with volatile bioactive substances (**B**), absorbent pads (**C**) and indicators wrapped around foods (**D**).

Meat products are nutritious but highly perishable. Meat spoilage is typically caused by microbial contamination, lipid peroxidation and autolytic enzymatic cleavage, which is always accompanied by the release of nitrogen-based volatile compounds [7]. In most existing studies, the films containing PSPAs are cut into small strips and used as indicators to sense the nitrogen-based volatile compounds of different meat foods, such as pork, beef, chicken, fish and shrimp (Table 2). The nitrogen-based volatile compounds can dose-dependently cause color changes in the films. Based on this principle, the freshness/spoilage degree of meat products can be predicted by the color of the films. In practical application, the tested meat products are normally stored in plastic trays that are stuck with the indicators inside the cap of the trays. During the whole storage period, the tested foods are kept no-touch with the indicators. Meanwhile, the plastic trays should be closely sealed to avoid the leakage of nitrogen-based volatile compounds (Figure 7A). In contrast, the application of the indicators by directly contacting meat products is not recommended because the migration of PSPAs into foods is inevitable [107].

Recently, dual-function smart packaging films with freshness-maintaining and freshness-indicating functions have been developed by some researchers. The preferential option is to add volatile substances (e.g., carvacrol and eugenol) with antioxidant and antibacterial activity into the indicators (Figure 7B), and the modified indicators can not only indicate meat freshness but also extend meat shelf life by releasing the volatile bioactives without contacting foods [90,105]. Thus, as compared with the films without volatile bioactive substances, the modified indicators show slower color changes in indicating meat freshness [90,105]. The second option is to utilize the films containing PSPAs as absorbent pads (Figure 7C), where meat products are directly placed on the top of pads during storage [86,104]. In this way, the bioactive PSPAs can be released into meat and exert a freshness-maintaining effect. Meanwhile, the pads can indicate meat freshness through the color changes of PSPAs in the pads [86,104]. The last option is to wrap the indicators around meat foods (Figure 7D), where the indicators can release PSPAs and other bioactives to exert freshness-indicating and freshness-maintaining functions [87,98,113]. It should be noted that, when the films are designed to directly contact meat products, the safety of the indicators needs to be evaluated beforehand. However, researchers have not realized the importance in evaluating the safety of the indicators. Meanwhile, the influence of the indicators on the environment (i.e., biodegradability) is seldom tested by researchers [87,96,102,111].

The effectiveness of the indicators is evaluated not only by observing the color changes of the indicators during meat storage but also by measuring the physiological and biochemical indexes of the foods, such as pH, aerobic plate counts, TBARS and TVB-N levels. Since the color changes of the indicators can be quantitatively analyzed by measuring CIE-Lab and RGB color coordinates, the relationship between the color changes of the indicators and the freshness/spoilage degree (e.g., fresh, medium-fresh and spoilage) of meat products is often analyzed. Researchers have demonstrated that the total color difference (Δ*E*) of the indicators has a high positive correlation with the TVB-N and TVC of meat products [85,94,97,100,102]. In addition, the G/R ratio of the indicators is positively correlated with the TVB-N of meat products [99,113]. It is worth noting principal components analysis (PCA) is a powerful tool to analyze the CIE-Lab and RGB color coordinates of the indicators [84,85,88]. For fresh, medium-fresh and spoiled foods, the feasible films should show color coordinates at different quadrants [84,88]. Therefore, the freshness/spoilage degree of meat products can be accurately predicted by measuring the color coordinates of the indicators. It is worth noting that the measurement of CIE-Lab color coordinates can only be performed by a specific colorimeter, which is not available to common consumers. In contrast, the measurement of RGB color coordinates can be realized by a smartphone, which is accessible to common consumers. Thus, the accurate prediction of meat freshness/spoilage degree by using RGB detection with a smartphone is more recommended in recent studies [88,99,113].

Except for indicating and maintaining the freshness of meat products, the indicators containing PSPSs can also be used in other fields. Two recent studies have revealed starch/gelatin/PSPA indictors are capable of monitoring the quality changes of enokitake and white oyster mushrooms during storage, which is based on the CO_2_-sensing ability of the indicators [110,118]. The indicators change from green to purplish gray, and finally, to yellowish green during the room temperature storage of the mushrooms. Unfortunately, correlation analysis was not conducted between the indicators’ color and the mushrooms’ quality (especially respiration rate) in these studies [110,118]. Recently, Yan et al. [115] found the red carboxymethyl cellulose/PSPA indicators gradually turned yellow after immersing them in sunflower seed oil with different oxidized degrees for 48 h. Moreover, a positive correlation was found between the Δ*E* changes of the indicators and the peroxide value of the oil, indicating that the indicators were applicable to monitoring the oxidative degree of edible oil. Despite this, this indication manner is not observable in real time, and thus this method is seldom applied by other researchers.

### 5.7. Limitations and Challenges

Although PSPAs have good antioxidant activity and color-changing ability, they have low antibacterial activity and stability. Therefore, additional antibacterial agents (e.g., essential oils, polyphenols and nanoparticles) and stabilizing agents (e.g., the suitable film matrix, other pigments and hydrophobic substances) are needed for enhancing the performance of smart packaging films containing PSPAs. It is worth noting that the content and composition of PSPAs are greatly associated with the cultivar and the source of PSP. As reported, there are more than 60 cultivars of PSP commonly planted around the world [78]. It is necessary to screen the suitable cultivar of PSP for the production of smart packaging films. Nowadays, the PSPAs used for the production of smart packaging films are normally isolated from the root of PSP. However, PSPAs from the leaves of PSP have not been utilized for the production of smart packaging films. Notably, the conventional extraction method of PSPAs requires a large amount of water and organic solvents, which are uneconomical and environmentally unfriendly. The development of solvent-saving extraction methods for PSPAs is urgent. The usage of the cooked PSP powder is another choice for producing smart packaging films in an economic and environmentally friendly way [89]. Since PSPAs are heat-sensitive substances, the production of smart packaging films based on PSPAs normally requires low-temperature operating conditions. However, the existing industrial equipment used for the production of petroleum-based plastic packaging films often requires high-temperature operating conditions, and thus is not suitable for the large-scale production of smart packaging films containing PSPAs. Thus, new industrial equipment needs to be designed for the large-scale production of smart packaging films containing PSPAs.

## 6. Conclusions

The natural colorants of PSPAs can be extracted from the roots or leaves of PSP by different means, including solvent extraction, ultrasound-assisted extraction, enzyme-assisted extraction, microwave-assisted extraction, pulsed electric field extraction, pressurized liquid extraction, high-pressure carbon dioxide extraction and their combinations. Then PSPAs can be isolated from the extract by ATPS, membrane separation and column chromatography, which are followed by composition characterization with HPLC-MS and a HPLC-UV detector as well as purification by semi-preparative HPLC and HSCCC. The extraction yield and composition of PSPAs are greatly affected by the extraction condition, extraction method, cultivar, part and pre-cooking of PSP. Since PSPAs have strong antioxidant activity, certain antimicrobial activities and superior pH-response color-changing ability, they are added into the biopolymer-based film matrix to develop smart packaging films through solvent casting, electrospinning and 3D-printing techniques. The physical and functional properties of the films are affected by the amount of PSPAs in the films, the cultivar and pre-cooking of PSP, the composition of the film matrix, the addition of reinforcing agents, and the preparation method and conditions of the films. Nowadays, the biopolymer-based films containing PSPAs are primarily used for indicating and maintaining the freshness of meat products. In the future, more efforts should be made on the purification of PSPAs and the functional and stability enhancements of biopolymer-based films containing PSPAs. The application of the films could be extended from meat products to other types of foods.

## Figures and Tables

**Table 1:** The extraction method, optimal extraction condition and maximum extraction yield of PSPAs.

Part of PSP	Extraction Method	Experimental Design Method	Optimal Extraction Condition	Maximum Extraction Yield	References
Flesh	Solvent extraction	Taguchi orthogonal design	Solid–liquid ratio: 1:10 (*w*/*v*) Solvent composition: 80% ethanol containing 0.1% HCl Extraction temperature: 60 °C Extraction time: 90 min	217.58 mg/100 g DW	[29]
Flesh	Ultrasound-assisted extraction	Taguchi orthogonal design	Solid–liquid ratio: 1:10 (*w*/*v*) Solvent composition: 90% ethanol containing 0.1% HCl Ultrasound temperature: 50 °C Ultrasound time: 45 min Ultrasonic power: 200 W	229.41 mg/100 g DW	[29]
Flesh	Pressurized liquid extraction	Taguchi orthogonal design	Solvent composition: 80% ethanol containing 0.1% HCl Extraction temperature: 90 °C Static time: 15 min Static cycle: 2	244.07 mg/100 g DW	[29]
Flesh	Ultrasound-assisted extraction	Box–Behnken design	Solid–liquid ratio: 1:42 (*w*/*v*) Solvent composition: 83% polyethylene glycol 200 Ultrasound temperature: 64 °C Ultrasound time: 80 min Ultrasonic power: 100 W Ultrasonic frequency: 40 KHz	83.78 mg/100 g DW	[35]
Flesh	Solvent extraction	Full factorial experimental design	Solid–liquid ratio: 1:30 (*w*/*v*) Solvent composition: 70% methanol containing 7% acetic acid Extraction temperature: 80 °C	186.10 mg/100 g FW	[38]
Flesh	Solvent extraction	Box–Behnken design	Solid–liquid ratio: 1:32 (*w*/*v*) Solvent composition: ethanol containing 1.5 mol/L HCl Extraction temperature: 80 °C Extraction time: 60 min	158.00 mg/100 g DW	[40]
Flesh	Solvent extraction	Box–Behnken design	Solid–liquid ratio: 1:30 (*w*/*v*) Solvent composition: 80% acidified ethanol Extraction temperature: 60 °C Extraction time: 60 min	1163.55 mg/100 g DW	[41]
Flesh	Solvent extraction	Face-centered cube design	Solvent composition: 1.38% citric acid Extraction temperature: 62.91 °C Extraction time: 2.53 min	19.78 mg/100 g FW	[42]
Flesh	Ultrasound-assisted extraction	Box–Behnken design	Solid–liquid ratio: 1:10 (*w*/*v*) Solvent composition: 58% ethanol pH: 2.5 Extraction temperature: 80 °C Extraction time: 120 min Ultrasound time: 40 min Ultrasonic power: 178 W Ultrasonic frequency: 45 KHz	29.30 mg/100 g FW	[44]
Flesh	Ultrasound-assisted enzymatic extraction	Box–Behnken design	Solid–liquid ratio: 1:36 (*w*/*v*) Solvent composition: 95% ethanol containing and 0.1% HCl (40:60, *v*/*v*) Extraction temperature: 60 °C Ultrasound time: 35 min Enzyme dosage: 2.35 mg/g solid	200.30 mg/100 g DW	[45]
Flesh	Ultrasound-assisted extraction	Central composite design	Solid–liquid ratio: 1:10 (*w*/*v*) Solvent composition: ethanol containing 1.0 mol/L HCl Ultrasound temperature: 25 °C Ultrasound time: 22 min Ultrasonic power: 150 W Ultrasonic frequency: 43 KHz	35.32 mg/100 g DW	[46]
Flesh	Ultrasound-assisted enzymatic extraction	Box–Behnken design	Solid–liquid ratio: 1:15 (*w*/*v*) Solvent composition: 78% ethanol solution Enzyme dosage: 1.0%, cellulase/pectinase/papain = 2:2:1 pH: 4.5 Enzymatic hydrolysis time: 41 °C Enzymatic hydrolysis temperature: 90 min Ultrasound temperature: 48 °C Ultrasound time: 20 min Ultrasound cycle: 2	227.00 mg/100 g DW	[47]
Flesh	Microwave-assisted extraction	Box–Behnken design	Solid–liquid ratio: 1:3 (*w*/*v*) Solvent composition: 30% ethanol containing 2 mL of 10% critic acid pH: 2 Microwave time: 3 min Microwave power: 320 W	31.16 mg/100 g FW	[48]
Flesh	Pressurized liquid extraction	Face-centered cube design	Solvent composition: acetic acid/methanol/water = 7:75:18 (*v*/*v*) Extraction temperature: 80–100 °C Extraction pressure: 1500 psi	663.00 mg/100 g DW	[49]

FW, Fresh weight; DW: dry weight.

**Table 2:** Formulations, physical and functional properties, and applications of smart packaging films based on biopolymers and PSPAs.

Cultivar of PSP	Film Matrix	Reinforcing Agents	Film Preparation Methods	Impacts of PSPAs on the Physical Properties of the Films	Functional Properties of the Films	Factors Affecting the Properties of the Films	Application of the Films	Cultivar of PSP
Unknown	Starch/PVA blend		Solvent casting	LT↓, TS↔, EB↑	Antioxidant activity (DPPH radical scavenging activity) pH sensitivity		Monitoring pork and shrimp freshness	[17]
Unknown	Agar/potato starch blend			LT↓	pH sensitivity		Monitoring pork freshness	[22]
Tainung 73	Gellan gum/PSP powder blend		Solvent casting	SR↓, WCA↑, WVP↑, TS↑, EB↑	Antioxidant activity (DPPH and ABTS radical scavenging activity) pH sensitivity	The content of anthocyanins	Monitoring *E. coli* growth	[23]
Unknown	Ethyl cellulose	Castor oil	Solvent casting	TS↓, EB↑	pH sensitivity Ammonia sensitivity	The presence of castor oil	Monitoring pork freshness	[47]
Unknown	Agar		3D-printing		Ammonia sensitivity		Monitoring beef and fish freshness	[83]
Unknown	Starch/PVA blend	Curcumin	Solvent casting	MC↔, WS↔, WVP↔	Ammonia sensitivity	The presence of curcumin	Monitoring fish freshness	[84]
Unknown	κ-Carrageenan	Betacyanins	Solvent casting	WS↑, WVP↔, WCA↓, LT↓, TS↔, EB↔	Antioxidant activity (DPPH and ABTS radical scavenging activity)	The ratio of anthocyanins and the presence of betacyanins	Monitoring pork freshness	[85]
Unknown	PVA/paper fiber blend	Silver nanoparticles	Freezing and thawing treatment and freeze-drying		Antioxidant activity (DPPH radical scavenging activity) Antimicrobial activity against *Salmonella* pH sensitivity	The presence of the biogenic silver nanoparticles	Monitoring pork freshness and extending pork shelf life	[86]
Unknown	PVA/chitosan blend	Zinc oxide nanoparticles	Solvent casting		Antioxidant activity (ABTS radical scavenging activity) Antimicrobial activity against *S. aureus* pH sensitivity	The presence of zinc oxide nanoparticles	Monitoring chicken freshness and extending chicken shelf life	[87]
Unknown	Indicator layer: agar Antibacterial layer: carrageenan	Tannic acid, oregano essential oil and silver nanoparticles	Solvent casting		Antioxidant activity (DPPH radical scavenging activity) Antimicrobial activity against *E. coli* and *S. aureus* pH sensitivity	The content of oregano essential oil Pickering emulsion and the presence of silver nanoparticles	Extending shelf life of beef	[88]
Guizishu 03	Cooked PSP powder/sodium alginate blend		Solvent casting		Antioxidant activity (DPPH radical scavenging activity) pH sensitivity	The type of cooking treatment	Monitoring shrimp freshness	[89]
Unknown	Cellulose acetate	Cyclodextrin metal-organic framework and eugenol	Solvent casting	WCA↓, WVP↓, OP↓, LT↓, TS↑, EB↓	Antioxidant activity (DPPH radical scavenging activity) Antimicrobial activity against *E. coli* and *S. aureus * Ammonia sensitivity	The encapsulation of PSPAs and the presence of eugenol	Monitoring pork freshness and extending pork shelf life	[90]
Unknown	Ethyl cellulose/gelatin blend		Electrospinning	WCA↑, WVP↓	Antioxidant activity (DPPH radical scavenging activity) pH sensitivity Ammonia sensitivity	The content of anthocyanins and film preparation method	Monitoring pork freshness	[91]
Unknown	Chitosan/PVA blend	Zinc oxide nanoparticles	Solvent casting			The presence of zinc oxide nanoparticles	Extending fish shelf life	[92]
Unknown	Starch or starch/ carboxymethyl cellulose blend		Solvent casting	WS↔, MC↓, LT↓, TS↓, EB↑	pH sensitivity	The content of anthocyanins	Monitoring beef freshness	[93]
Guizi 3	Steamed PSP powder/sodium alginate blend	Mandarin essential oil	Solvent casting	WVP↓, OP↑, TS↓, EB↓	Antioxidant activity (DPPH radical scavenging activity) Antimicrobial activity against *E. coli* and *S. aureus * pH sensitivity	The content of mandarin essential oil	Monitoring shrimp and pork freshness	[94]
Fuzi 1, Ganzi 6, Jizi 3, Ningzi 2, Ningzi 4, Qining 2, Qining 18, Xiangzi 910, Xuzi 8	Steamed PSP powder/sodium alginate blend	Mandarin essential oil	Solvent casting		pH sensitivity Ammonia sensitivity	The cultivar of PSP	Monitoring shrimp freshness	[95]
Unknown	Gelatin	Oxidized alginate	Solvent casting	SR↓, WCA↔, WVP↔, LT↓, TS↑, EB↑	Antioxidant activity (DPPH radical scavenging activity) pH sensitivity Ammonia sensitivity Biodegradability	The content of oxidized alginate	Monitoring fish, chicken and pork freshness	[96]
Unknown	Carboxymethyl cellulose/starch blend		Solvent casting	MC↓, LT↓, TS↑, EB↓	pH sensitivity Ammonia sensitivity	The content of anthocyanins	Monitoring fish freshness	[97]
Unknown	Sodium alginate		Solvent casting		pH sensitivity Ammonia sensitivity	The drying temperature of the films		[98]
Unknown	PVA/sodium alginate blend	Modified aramid nanofiber and CaCl_2_	Freezing–thawing treatment and immersing		Ammonia sensitivity	The presence of modified aramid nanofiber	Monitoring shrimp freshness	[99]
Unknown	Sodium alginate	CaCl_2_ (cross-linking agent)	Solvent casting	WVP↓, LT↓, TS↓, EB↓	pH sensitivity Ammonia sensitivity	The content of anthocyanins	Monitoring chicken freshness	[100]
Unknown	Agarose		Solvent casting		pH sensitivity Ammonia sensitivity	The content of anthocyanins, the pH of film-forming solution and relative humidity		[101]
Unknown	PVA/riclin blend		Solvent casting	WCA↔, WVP↑, OP↓, LT↓, TS↓, EB↑	Antioxidant activity (OH, DPPH and ABTS radical scavenging activity) pH sensitivity Ammonia sensitivity Biodegradability	The presence of riclin and the content of anthocyanins	Monitoring shrimp freshness	[102]
Unknown	Indicator layer: carrageenan/pectin blend Antibacterial layer: carrageenan	Magnolol	Solvent casting	WVP↑, TS↓, EB↓	Antioxidant activity (DPPH radical scavenging activity) Antimicrobial activity against *E. coli* and *S. aureus * pH sensitivity Ammonia sensitivity	The presence of magnolol	Monitoring fish freshness	[103]
Unknown	PVA/agarose blend		Solvent casting	WS↑, SR↑, WCA↓, TS↓	Antioxidant activity (DPPH radical scavenging activity) pH sensitivity	The content of anthocyanins	Monitoring pork freshness	[104]
Unknown	Indicator layer: pullulan Antibacterial layer: zein	Carvacrol	Electrospinning		Antimicrobial activity against *E. coli* and *S. aureus * Antioxidant activity (DPPH radical scavenging activity) Ammonia and acetic acid sensitivity	The content of anthocyanins and the presence of carvacrol	Monitoring pork freshness	[105]
Unknown	Ethyl cellulose/gelatin blend	ε-Polylysine	Electrospinning		Antioxidant activity (DPPH radical scavenging activity) Antimicrobial activity against *E. coli* and *S. aureus * pH sensitivity Ammonia sensitivity	The ratio of film matrix, the film preparation method and the presence of ε-polylysine	Monitoring pork freshness and extending pork shelf life	[106]
Anggun 1	PSP starch, PSP starch/PSP powder blend		Solvent casting	MC↔, WS↑, SR↑, WVP↓, TS↓, EB↑	pH sensitivity	The nature of the film matrix and the content of anthocyanins	Monitoring chicken freshness	[107]
Xuzi 8	Chitosan		Solvent casting	MC↓, WS↑, WVP↑, LT↓, TS↓, EB↓	Antioxidant activity (DPPH radical scavenging activity) pH sensitivity	The content of anthocyanins		[108]
Unknown	Locust bean gum/PVA blend, chitosan/PVA blend, κ-carrageenan/PVA blend		Solvent casting	WVP↔, TS↔, EB↓	Antioxidant activity (DPPH radical scavenging activity) pH sensitivity Ammonia sensitivity	The type of film matrix	Monitoring shrimp freshness	[109]
Unknown	Starch/gelatin blend		Solvent casting			The ratio of starch and gelatin	Monitoring *Flammulina velutipes* mushroom freshness	[110]
Unknown	PVA/sodium alginate/sodium carboxymethyl cellulose blend		Solvent casting	MC↔, WCA↓, WVP↑, LT↓, TS↔, EB↓	Antioxidant activity (DPPH and ABTS radical scavenging activity) pH sensitivity	The content of anthocyanins	Monitoring pork freshness and extending cherry shelf life	[111]
Unknown	Cellulose nanofiber	Oregano essential oil	Solvent casting	WCA↓, LT↓, TS↓, EB↑	Antimicrobial activity against *E. coli* and *L. monocytogenes* pH sensitivity	The presence of oregano essential oil		[112]
Unknown	Agar/sodium alginate blend	Quercetin-loaded chitosan nanoparticles	Solvent casting	WS↔, WVP↔, OP↔, LT↓, TS↑, EB↑	Antioxidant activity (DPPH radical scavenging activity) Antimicrobial activity against *E. coli* and *S. aureus * pH sensitivity Ammonia sensitivity	The presence of quercetin-loaded chitosan nanoparticles	Monitoring shrimp freshness and extending shrimp shelf life	[113]
Unknown	Chitosan/hydroxypropyl methylcellulose blend	ε-Polylysine	Solvent casting	MC↓, WVP↔, LT↓, TS↑, EB↑	Antimicrobial activity against *E. coli* and *S. aureus * pH sensitivity	The presence of ε-polylysine	Monitoring fish freshness	[114]
Unknown	Corn starch/carboxymethyl cellulose blend		Solvent casting	MC↓, LT↓, TS↑, EB↑	Hydrogen peroxide sensitivity	The content of anthocyanins	Monitoring the oxidation degree of sunflower seed oil	[115]
Unknown	Corn starch/PVA blend		Solvent casting	WS↑, WVP↑, LT↓, TS↑, EB↑	pH sensitivity	The content of anthocyanins	Monitoring shrimp freshness	[116]
Unknown	Chitosan/PVA blend		Solvent casting	MC↔, WS↔, WCA↓, LT↓	pH sensitivity		Monitoring beef freshness	[117]
Unknown	Chitosan/corn starch blend		Solvent casting and immersing	MC↓, WS↓, SR↓, TS↑	pH sensitivity		Monitoring mushroom freshness	[118]

ABTS, 2,2′-azino-bis(3-ethylbenzothiazoline-6-sulfonate); DPPH, 2,2-diphenyl-1-picrylhydrazyl; EB, elongation at break; LT, light transmittance; MC, moisture content; OP, oxygen permeability; PSP, purple sweet potato; PSPAs, purple sweet potato anthocyanins; PVA, polyvinyl alcohol; SR, swelling ratio; TS, tensile strength; WCA, water contact angle; WS, water solubility; WVP, water vapor permeability; ↑, increased after PSPA addition; ↓, decreased after PSPA addition; ↔, unchanged after PSPA addition.

## Data Availability

The original contributions presented in this study are included in the article/Supplementary Materials. Further inquiries can be directed to the corresponding author.

## Supplementary Materials

The following supporting information can be downloaded at: www.mdpi.com/article/10.3390/foods13213485/s1, Table S1: The composition of PSPAs affected by the extraction method, cultivar, part and pre-cooking of PSP.
