# Supplementary material for: Recent Advances in Purple Sweet Potato Anthocyanins: Extraction, Isolation, Functional Properties and Applications in Biopolymer-Based Smart Packaging
Source: Foods. 2024 Oct 30;13(21):3485. doi: 10.3390/foods13213485 (PMC11545044; doi:10.3390/foods13213485)
Supplement: Supplementary file 1 [file foods-13-03485-s001.zip › foods-3260080-supplementary.pdf]

Table S1

The composition of PSPAs affected by the extraction method, cultivar, part and pre-cooking of PSP.

Part	Cultivar	Composition of anthocyanins	References
Root		**Conventional extraction**: cyanidin-3-sophoroside-5-glucoside (4.4%), peonidin-3-sophoroside-5-glucoside (9.0%), cyanidin-3-*p*-hydroxybenzoyl-sophoroside-5-glucoside (4.1%), peonidin-3-*p*-hydroxybenzoyl-sophoroside-5-glucoside (1.0%), cyanidin-3-feruloyl-sophoroside-5-glucoside (2.9%), cyanidin-3-caffeoyl-sophoroside-5-glucoside (14.0%), peonidin-3-feruloyl-sophoroside-5-glucoside (6.2%), peonidin-3-caffeoyl-sophoroside-5-glucoside (1.7%), cyanidin-3-caffeoyl-*p*-hydroxybenzoyl-sophoroside-5-glucoside (9.7%), cyanidin-3-caffeoyl-feruloyl-sophoroside-5-glucoside (1.3%), peonidin-3-caffeoyl-*p*-hydroxybenzoyl-sophoroside-5-glucoside (28.1%), peonidin-3-caffeoyl-feruloyl-sophoroside-5-glucoside (17.6%)<br>**Ultrasound-assisted extraction**: cyanidin-3-sophoroside-5-glucoside (4.0%), peonidin-3-sophoroside-5-glucoside (10.1%), cyanidin-3-*p*-hydroxybenzoyl-sophoroside-5-glucoside (4.3%), peonidin-3-*p*-hydroxybenzoyl-sophoroside-5-glucoside (0.8%), cyanidin-3-feruloyl-sophoroside-5-glucoside (1.2%), cyanidin-3-caffeoyl-sophoroside-5-glucoside (13.4%), peonidin-3-feruloyl-sophoroside-5-glucoside (6.5%), peonidin-3-caffeoyl-sophoroside-5-glucoside (1.9%), cyanidin-3-caffeoyl-*p*-hydroxybenzoyl-sophoroside-5-glucoside (10.6%), cyanidin-3-caffeoyl-feruloyl-sophoroside-5-glucoside (1.4%), peonidin-3-caffeoyl-*p*-hydroxybenzoyl-sophoroside-5-glucoside (28.9%), peonidin-3-caffeoyl-feruloyl-sophoroside-5-glucoside (16.9%)<br>**Accelerated-solvent extraction**: cyanidin-3-sophoroside-5-glucoside (2.0%), peonidin-3-sophoroside-5-glucoside (5.9%), cyanidin-3-*p*-hydroxybenzoyl-sophoroside-5-glucoside (4.2%), peonidin-3-*p*-hydroxybenzoyl-sophoroside-5-glucoside (0.5%), cyanidin-3-feruloyl-sophoroside-5-glucoside (0.8%), cyanidin-3-caffeoyl-sophoroside-5-glucoside	[29]

		(11.2%), peonidin-3-feruloyl-sophoroside-5-glucoside (3.7%), peonidin-3-caffeoyl-sophoroside-5-glucoside (2.4%), cyanidin-3-caffeoyl-*p*-hydroxybenzoyl-sophoroside-5-glucoside (15.8%), cyanidin-3-caffeoyl-feruloyl-sophoroside-5-glucoside (1.4%), peonidin-3-caffeoyl-*p*-hydroxybenzoyl sophoroside-5-glucoside (24.8%), peonidin-3-caffeoyl-feruloyl-sophoroside-5-glucoside (27.2%)	
Root		Cyanidin-3-sophoroside-5-glucoside (3.16%), cyaniding-3-*p*-hydroxybenzoyl-sophoroside-5-glucoside (13.46%), cyanidin-3-feruloyl-sophoroside-5-glucoside (2.31%), cyaniding-3-caffeoyl-sophoroside-5-glucoside (2.78%), cyaniding-3-caffeoyl-*p*-hydroxybenzoyl-sophoroside-5-glucoside (16.79%), peonidin-3-caffeoyl-sophoroside-5-glucoside (49.1%), cyaniding-3-caffeoyl-feruloyl-sophoroside-5-glucoside (6.34%), peonidin-3-caffeoyl-*p*-hydroxybenzoyl-sophoroside-5-glucoside (6.07%)	[24]
Root	Provenances 1, 2 and 3	**Provenances 1**: cyaniding-3-sophoroside-5-glucoside, peonidin-3-sophoroside-5-glucoside, cyaniding-3-*p*-hydroxybenzoyl-sophoroside-5-glucoside, cyaniding-3-caffeoyl-sophoroside-5-glucoside, peonidin-3-*p*-hydroxybenzoyl-sophoroside-5-glucoside, peonidin-3-caffeoyl-sophoroside-5-glucoside, cyaniding-3-feruloyl-sophoroside-5-glucoside, peonidin-3-feruloyl-sophoroside-5-glucoside, cyaniding-3-dicaffeoyl-sophoroside-5-glucoside, cyaniding-3-caffeoyl-*p*-hydroxybenzoyl-sophoroside-5-glucoside, cyaniding-3-caffeoyl-sophoroside-5-glucoside, cyaniding-3-caffeoyl-feruloyl-sophoroside-5-glucoside, peonidin-3-dicaffeoyl-sophoroside-5-glucoside, peonidin-3-caffeoyl-*p*-hydroxybenzoyl-sophoroside-5-glucoside, peonidin-3-caffeoyl-feruloyl-sophoroside-5-glucoside, peonidin-3-caffeoyl-*p*-coumaroyl-sophoroside-5-glucoside, pelargonidin-3-caffeoyl-feruloyl-sophoroside-5-glucoside, peonidin-3-feruloyl-*p*-hydroxybenzoyl-sophoroside-5-glucoside<br>**Provenances 2**: cyaniding-3-sophoroside-5-glucoside, peonidin-3-sophoroside-5-glucoside, cyanidin-3-*p*-hydroxybenzoyl-sophoroside-5-glucoside, cyanidin-3-caffeoyl-sophoroside-5-glucoside, peonidin-3-*p*-hydroxybenzoyl-sophoroside-5-glucoside, peonidin-3-caffeoyl-sophoroside-5-glucoside, cyanidin-3-feruloyl-sophoroside-5-glucoside, peonidin-3-feruloyl-sophoroside-5-glucoside, cyanidin-3-dicaffeoyl-sophoroside-5-glucoside, cyanidin-3-caffeoyl-*p*-hydroxybenzoyl-sophoroside-5-glucoside, cyanidin-3-caffeoyl-sop	[60]

		horoside-5-glucoside, cyanidin-3-caffeoyl-feruloyl-sophoroside-5-glucoside, peonidin-3-dicaffeoyl-sophoroside-5-glucoside, peonidin-3-caffeoyl-*p*-hydroxybenzoyl-sophoroside-5-glucoside, peonidin-3-caffeoyl-feruloyl-sophoroside-5-glucoside, peonidin-3-caffeoyl-*p*-coumaroyl-sophoroside-5-glucoside, pelargonidin-3-caffeoyl-feruloyl-sophoroside-5-glucoside, peonidin-3-feruloyl-*p*-hydroxybenzoyl-sophoroside-5-glucoside, peonidin-3--feruloyl-*p*-coumaroyl-sophoroside-5-glucoside<br>**Provenances 3**: cyanidin-3-sophoroside-5-glucoside, peonidin-3-sophoroside-5-glucoside, cyanidin-3-*p*-hydroxybenzoyl-sophoroside-5-glucoside, cyanidin-3-caffeoyl-sophoroside-5-glucoside, peonidin-3-*p*-hydroxybenzoyl-sophoroside-5-glucoside, peonidin-3-caffeoyl-sophoroside-5-glucoside, cyanidin-3-feruloyl-sophoroside-5-glucoside, peonidin-3-feruloyl-sophoroside-5-glucoside, cyanidin-3-dicaffeoyl-sophoroside-5-glucoside, cyanidin-3-caffeoyl-*p*-hydroxybenzoyl-sophoroside-5-glucoside, cyanidin-3-caffeoyl-sophoroside-5-glucoside, cyanidin-3-caffeoyl-feruloyl-sophoroside-5-glucoside, peonidin-3-dicaffeoyl-sophoroside-5-glucoside, peonidin-3-caffeoyl-*p*-hydroxybenzoyl-sophoroside-5-glucoside, peonidin-3-caffeoyl-feruloyl-sophoroside-5-glucoside, peonidin-3-caffeoyl-*p*-coumaroyl-sophoroside-5-glucoside	
Root	Sinjami and Yeonjami	Cyanidin-3-*p*-hydroxybenzoyl-sophoroside-5-glucoside, peonidin-3-p-hydroxybenzoyl-sophoroside-5-glucoside, cyanidin-3-feruloyl-sophoroside-5-glucoside, peonidin-3-feruloyl-sophoroside-5-glucoside, cyanidin-3-caffeoyl-*p*-hydroxybenzoyl-sophoroside-5-glucoside, peonidin-3-caffeoyl-*p*-hydroxybenzoyl-sophoroside-5-glucoside	[25]
Root	6-6-12, 6-6-16, 6-8-37, 6-12-2, Yuzishu-3a, Yuzishu-7a, 6-13-32, Qianzishu-1a, 6-21-27, 6-24-50, 7-11-8, 7-12-21, 7-28-2, 7-28-10, 7-33-2, 7-33-15, 7-35-2, 7-44-10, 8-3-3, 8-4-11, 8-13-28, 8-13-4	Cyanidin-3-sophoroside-5-glucoside, peonidin-3-sophoroside-5-glucoside, cyanidin-3-*p*-hydroxybenzoyl-sophoroside-5-glucoside, peonidin-3-*p*-hydroxybenzoyl-sophoroside-5-glucoside, cyanidin-3-feruloyl-sophoroside-5-glucoside, peonidin-3-feruloyl-sophoroside-5-glucoside, cyanidin-3-caffeoyl-sophoroside-5-glucoside, cyanidin-3-caffeoyl-*p*-hydroxybenzoyl-sophoroside-5-glucoside, cyanidin-3-caffeoyl-feruloyl-sophoroside-5-glucoside, peonidin-3-caffeoyl-sophoroside-5-glucoside, peonidin-3-dicaffeoyl-sophoroside-5-glucoside, peonidin-3-caffeoyl-*p*-hydroxybenzoyl-sophoroside-5-glucoside, peonidin-3	[61]

	9, 8-24-15, 8-33-5, Rizishu-7a, Rizishu-13a, 8-45-9, Wanzishu-56a, Yusuzi-43a and Yuzishu-263a	-caffeoyl-feruloyl-sophoroside-5-glucoside	
Root	Yamakawamurasaki	Cyanidin-3-sophoroside-5-glucoside (3.5%), peonidin-3-sophoroside-5-glucoside (3.8%), cyanidin-3-*p*-hydroxybenzoyl-sophoroside-5-glucoside (2.9%), peonidin-3-*p*-hydroxybenzoyl-sophoroside-5-glucoside (4.3%), cyanidin-3-feruloyl-sophoroside-5-glucoside (4.8%), cyanidin-3-caffeoyl-sophoroside-5-glcoside (3.9%), cyanidin-3-caffeoyl-*p*-hydroxybenzoyl-sophoroside-5-glucoside (25.9%), peonidin-3-caffeoyl-*p*-hydroxybenzoyl-sophoroside-5-glucoside (14.9%), peonidin-3-caffeoyl-sophoroside-5-glucoside (12.6%), peonidin-3-caffeoyl-feruloyl-sophoroside-5-glucoside (4.7%)	[35]
Root (inner layer and outer layer)	Sinjami, Jami, Danjami, Yeonjami and Borami	**Outer layer of Sinjami:** cyanidin-3-sophoroside-5-glucoside (0.22%), peonidin-3-sophoroside-5-glucoside (1.21%), cyanidin-3-*p*-hydroxybenzoyl-sophoroside-glucoside (1.6%), cyanidin-3-caffeoyl-sophoroside-5-glucoside (0.24%), peonidin-3-*p*-hydroxybenzoyl-sophoroside-5-glucoside (8.78%), peonidin-3-caffeoyl-sophoroside-5-glucoside (1.38%), cyanidin-3-feruloyl-sophoroside-5-glucoside (1.68%), peonidin-3-feruloyl-sophoroside-5-glucoside (9.78%), cyanidin-3-caffeoyl-sophoroside-5-glucoside (0.24%), cyanidin-3-caffeoyl-*p*-hydroxybenzoyl-sophoroside-5-glucoside (6.13%), peonidin-3-caffeoyl-sophoroside-5-glucoside (0.02%), cyanidin-3-caffeoyl-feruloyl-sophoroside-5-glucoside (2.9%), peonidin-3-dicaffeoyl-sophoroside-5-glucoside (2.22%), peonidin-3-caffeoyl-*p*-hydroxybenzoyl-sophoroside-5-glucoside (40.62%), peonidin-3-caffeoyl-feruloyl-sophoroside-5-glucoside (22.46%), peonidin-3-feruloyl-*p*-hydroxybenzoyl-sophoroside-5-glucoside (0.5%)<br>**Outer layer of Danjami:** cyanidin-3-sophoroside-5-glucoside (0.97%), peonidin-3-sophoroside-5-glucoside (2.18%), cyanidin-3-*p*-hydroxybenzoyl-sophoroside-glucoside (3.31%), cyanidin-3-caffeoyl-sophoroside-5-glucoside (0.24%), peonidin-3-*p*-hydroxybenzoyl-sophoroside-5-glucoside (13.29%), peonidin-3-caffeoyl-sophoroside-5-glucoside	[64]

(0.39%), cyanidin-3-feruloyl-sophoroside-5-glucoside (0.73%), peonidin-3-feruloyl-sophoroside-5-glucoside (1.53%), cyanidin-3-caffeoyl-sophoroside-5-glucoside (0.39%), cyanidin-3-caffeoyl-*p*-hydroxybenzoyl-sophoroside-5-glucoside (14.58%), peonidin-3-caffeoyl-sophoroside-5-glucoside (0.06%), cyanidin-3-caffeoyl-feruloyl-sophoroside-5-glucoside (1.91%), peonidin-3-dicaffeoyl-sophoroside-5-glucoside (7.08%), peonidin-3-caffeoyl-*p*-hydroxybenzoyl-sophoroside-5-glucoside (47.02%), peonidin-3-caffeoyl-feruloyl-sophoroside-5-glucoside (5.86%), peonidin-3-feruloyl-*p*-hydroxybenzoyl-sophoroside-5-glucoside (0.05%)

Outer layer of Yeonjami: cyanidin-3-sophoroside-5-glucoside (0.81%), peonidin-3-sophoroside-5-glucoside (4.91%), cyanidin-3-*p*-hydroxybenzoyl-sophoroside-glucoside (1.67%), cyanidin-3-caffeoyl-sophoroside-5-glucoside (0.19%), peonidin-3-*p*-hydroxybenzoyl-sophoroside-5-glucoside (10.06%), peonidin-3-caffeoyl-sophoroside-5-glucoside (0.86%), cyanidin-3-feruloyl-sophoroside-5-glucoside (1.61%), peonidin-3-feruloyl-sophoroside-5-glucoside (7.67%), cyanidin-3-caffeoyl-sophoroside-5-glucoside (0.07%), cyanidin-3-caffeoyl-*p*-hydroxybenzoyl-sophoroside-5-glucoside (6.37%), peonidin-3-caffeoyl-sophoroside-5-glucoside (0.06%), cyanidin-3-caffeoyl-feruloyl-sophoroside-5-glucoside (2.63%), peonidin-3-dicaffeoyl-sophoroside-5-glucoside (6.65%), peonidin-3-caffeoyl-*p*-hydroxybenzoyl-sophoroside-5-glucoside (35.66%), peonidin-3-caffeoyl-feruloyl-sophoroside-5-glucoside (20.53%), peonidin-3-feruloyl-*p*-hydroxybenzoyl-sophoroside-5-glucoside (0.29%)

Outer layer of Jami: cyanidin-3-sophoroside-5-glucoside (0.27%), peonidin-3-sophoroside-5-glucoside (0.02%), cyanidin-3-*p*-hydroxybenzoyl-sophoroside-glucoside (0.66%), cyanidin-3-caffeoyl-sophoroside-5-glucoside (0.4%), peonidin-3-*p*-hydroxybenzoyl-sophoroside-5-glucoside (0.98%), peonidin-3-caffeoyl-sophoroside-5-glucoside (0.43%), cyanidin-3-feruloyl-sophoroside-5-glucoside (0.96%), peonidin-3-feruloyl-sophoroside-5-glucoside (0.9%), cyanidin-3-caffeoyl-sophoroside-5-glucoside (0.32%), cyanidin-3-caffeoyl-*p*-hydroxybenzoyl-sophoroside-5-glucoside (19.29%), peonidin-3-caffeoyl-sophoroside-5-glucoside (0.14%), cyanidin-3-caffeoyl-feruloyl-sophoroside-5-glucosi

de (12.46%), peonidin-3-dicaffeoyl-sophoroside-5-glucoside (0.95%), peonidin-3-caffeoyl-*p*-hydroxybenzoyl-sophoroside-5-glucoside (36.41%), peonidin-3-caffeoyl-feruloyl-sophoroside-5-glucoside (24.85%), peonidin-3-feruloyl-*p*-hydroxybenzoyl-sophoroside-5-glucoside (0.92%)

Outer layer of Borami: cyanidin-3-sophoroside-5-glucoside (0.07%), pelargonidin-3-sophoroside-5-glucoside (0.51%), peonidin-3-sophoroside-5-glucoside (0.53%), cyanidin-3-*p*-hydroxybenzoyl-sophoroside-glucoside (0.11%), cyanidin-3-caffeoyl-sophoroside)-5-glucoside (0.1%), peonidin-3-*p*-hydroxybenzoyl-sophoroside-5-glucoside (0.82%), peonidin-3-caffeoyl-sophoroside-5-glucoside (1.57%), pelargonidin-3-caffeoyl-sophoroside-5-glucoside (0.11%), peonidin-3-feruloyl-sophoroside-5-glucoside (0.97%), pelargonidin-3-feruloyl-sophoroside-5-glucoside (0.78%), cyanidin-3-caffeoyl-*p*-hydroxybenzoyl-sophoroside-5-glucoside (5.8%), cyanidin-3-caffeoyl-feruloyl-sophoroside-5-glucoside (6.51%), peonidin-3-dicaffeoyl-sophoroside-5-glucoside (1.86%), peonidin-3-caffeoyl-*p*-hydroxybenzoyl-sophoroside-5-glucoside (44.12%), peonidin-3-caffeoyl-feruloyl-sophoroside-5-glucoside (20.74%), pelargonidin-3-caffeoyl-feruloyl-sophoroside-5-glucoside (14.48%), peonidin-3-feruloyl-*p*-hydroxybenzoyl-sophoroside-5-glucoside (0.89%)

Inner layer of Sinjami: cyanidin-3-sophoroside-5-glucoside (0.19%), peonidin-3-sophoroside-5-glucoside (1.07%), cyanidin-3-*p*-hydroxybenzoyl-sophoroside-glucoside (1.12%), cyanidin-3-caffeoyl sophoroside-5-glucoside (0.17%), peonidin-3-*p*-hydroxybenzoyl-sophoroside-5-glucoside (9.19%), peonidin-3-caffeoyl sophoroside-5-glucoside (1.22%), cyanidin-3-feruloyl-sophoroside-5-glucoside (1.35%), peonidin-3-feruloyl-sophoroside-5-glucoside (8.86%), cyanidin-3-caffeoyl-sophoroside-5-glucoside (0.27%), cyanidin-3-caffeoyl-*p*-hydroxybenzoyl-sophoroside-5-glucoside (4.81%), peonidin-3-caffeoyl-sophoroside-5-glucoside (0.01%), cyanidin-3-caffeoyl-feruloyl-sophoroside-5-glucoside (2.71%), peonidin-3-dicaffeoyl-sophoroside-5-glucoside (3.04%), peonidin-3-caffeoyl-*p*-hydroxybenzoyl-sophoroside-5-glucoside (42.91%), peonidin-3-caffeoyl-feruloyl-sophoroside-5-glucoside (22.65%), peonidin-3-feruloyl-*p*-hydroxybenzoyl-sophorosid

e-5-glucoside (0.41%)

Inner layer of Danjami: cyanidin-3-sophoroside-5-glucoside (1.08%), peonidin-3-sophoroside-5-glucoside (2.42%), cyanidin-3-*p*-hydroxybenzoyl-sophoroside-glucoside (4.08%), cyanidin-3-caffeoyl sophoroside-5-glucoside (0.13%), peonidin-3-*p*-hydroxybenzoyl-sophoroside-5-glucoside (15.96%), peonidin-3-caffeoyl-sophoroside-5-glucoside (0.44%), cyanidin-3-feruloyl-sophoroside-5-glucoside (0.39%), peonidin-3-feruloyl-sophoroside-5-glucoside (0.72%), cyanidin-3-caffeoyl-sophoroside-5-glucoside (0.53%), cyanidin-3-caffeoyl-*p*-hydroxybenzoyl-sophoroside-5-glucoside (15.77%), peonidin-3-caffeoyl-sophoroside-5-glucoside (0.01%), cyanidin-3-caffeoyl-feruloyl-sophoroside-5-glucoside (0.83%), peonidin-3-dicaffeoyl-sophoroside-5-glucoside (8.99%), peonidin-3-caffeoyl-*p*-hydroxybenzoyl-sophoroside-5-glucoside (46.33%), peonidin-3-caffeoyl-feruloyl-sophoroside-5-glucoside (2.11%), peonidin-3-feruloyl-*p*-hydroxybenzoyl-sophoroside-5-glucoside (0.13%)

Inner layer of Yeonjami: cyanidin-3-sophoroside-5-glucoside (1.03%), peonidin-3-sophoroside-5-glucoside (5.28%), cyanidin-3-*p*-hydroxybenzoyl-sophoroside-glucoside (1.9%), cyanidin-3-caffeoyl-sophoroside-5-glucoside (0.08%), peonidin-3-*p*-hydroxybenzoyl-sophoroside-5-glucoside (13.42%), peonidin-3-caffeoyl-sophoroside-5-glucoside (0.5%), cyanidin-3-feruloyl-sophoroside-5-glucoside (0.85%), peonidin-3-feruloyl-sophoroside-5-glucoside (4.37%), cyanidin-3-caffeoyl-sophoroside-5-glucoside (0.17%), cyanidin-3-caffeoyl-*p*-hydroxybenzoyl-sophoroside-5-glucoside (8.03%), cyanidin-3-caffeoyl-feruloyl-sophoroside-5-glucoside (1.56%), peonidin-3-dicaffeoyl sophoroside-5-glucoside (12.23%), peonidin-3-caffeoyl-*p*-hydroxybenzoyl-sophoroside-5-glucoside (40.86%), peonidin-3-caffeoyl-feruloyl-sophoroside-5-glucoside (9.53%), peonidin-3-feruloyl-*p*-hydroxybenzoyl-sophoroside-5-glucoside (0.16%)

Inner layer of Jami: cyanidin-3-sophoroside-5-glucoside (0.18%), cyanidin-3-*p*-hydroxybenzoyl-sophoroside-glucoside (0.83%), cyanidin-3-caffeoyl-sophoroside-5-glucoside (0.31%), peonidin-3-*p*-hydroxybenzoyl-sophoroside-5-glucoside (0.65%), peonidin-3-caffeoyl-sophoroside-5-glucoside (0.23%), cyanidin-3-feruloyl-sophoroside-5-glucosi

		de (1.06%), peonidin-3-feruloyl-sophoroside-5-glucoside (0.43%), cyanidin-3-caffeoyl-sophoroside-5-glucoside (0.98%), cyanidin-3-caffeoyl-*p*-hydroxybenzoyl-sophoroside-5-glucoside (23.63%), peonidin-3-caffeoyl-sophoroside-5-glucoside (0.28%), cyanidin-3-caffeoyl-feruloyl-sophoroside-5-glucoside (12.87%), peonidin-3-dicaffeoyl-sophoroside-5-glucoside (1.19%), peonidin-3-caffeoyl-*p*-hydroxybenzoyl-sophoroside-5-glucoside (34.17%), peonidin-3-caffeoyl-feruloyl-sophoroside-5-glucoside (22.13%), peonidin-3-feruloyl-*p*-hydroxybenzoyl-sophoroside-5-glucoside (1.04%)<br>**Inner layer of Borami**: cyanidin-3-sophoroside-5-glucoside (0.12%), pelargonidin-3-sophoroside-5-glucoside (1.13%), peonidin-3-sophoroside-5-glucoside (1.64%), cyanidin-3-*p*-hydroxybenzoyl-sophoroside-glucoside (0.07%), cyanidin-3-caffeoyl-sophoroside-5-glucoside (0.16%), peonidin-3-*p*-hydroxybenzoyl-sophoroside-5-glucoside (1.92%), peonidin-3-caffeoyl-sophoroside-5-glucoside (2.8%), pelargonidin-3-caffeoyl-sophoroside-5-glucoside (0.12%), peonidin-3-feruloyl-sophoroside-5-glucoside (1.32%), pelargonidin-3-feruloyl-sophoroside-5-glucoside (0.72%), cyanidin-3-caffeoyl-sophoroside-5-glucoside (0.05%), cyanidin-3-caffeoyl-*p*-hydroxybenzoyl-sophoroside-5-glucoside (3.8%), cyanidin-3-caffeoyl-feruloyl-sophoroside-5-glucoside (6.48%), peonidin-3-dicaffeoyl-sophoroside-5-glucoside (5.2%), peonidin-3-caffeoyl-*p*-hydroxybenzoyl-sophoroside-5-glucoside (42.91%), peonidin-3-caffeoyl-feruloyl-sophoroside-5-glucoside (18.56%), pelargonidin-3-caffeoyl-feruloyl-sophoroside-5-glucoside (12.07%), peonidin-3-feruloyl-*p*-hydroxybenzoyl-sophoroside-5-glucoside (0.88%)	
Root	Shinzami	**Raw PSP**: cyanidin-3-sophoroside-5-glucoside (0.48%), peonidin-3-sophoroside-5-glucoside (1.45%), cyanidin-3-*p*-hydroxybenzoyl-sophoroside-5-glucoside (4.16%), peonidin-3-*p*-hydroxybenzoyl-sophoroside-5-glucoside (11.03%), cyanidin-3-feruloyl-sophoroside-5-glucoside (1.52%), cyanidin-3-caffeoyl-sophoroside-5-glucoside (1.27%), peonidin-3-feruloyl-sophoroside-5-glucoside (3.48%), cyanidin-3-dicaffeoyl-sophoroside-5-glucoside (1.65%), cyanidin-3-caffeoyl-*p*-hydroxybenzoyl-sophoroside-5-glucoside (10.21%), peonidin-3-caffeoyl-sophoroside-5-glucoside (5.51%), cyanidin-3-caffeoyl-feruloyl-sophoroside-5-glucoside (3.32%), peonidin-3-dicaffeoyl-sophoroside-5-glucoside	[26]

(3.33%), peonidin-3-caffeoyl-*p*-hydroxybenzoyl-sophoroside-5-glucoside (42.18%), peonidin-3-caffeoyl-feruloyl-sophoroside-5-glucoside (9.84%), peonidin-3-feruloyl-*p*-hydroxybenzoyl-sophoroside-5-glucoside (0.62%)

Steamed PSP: cyanidin-3-sophoroside-5-glucoside (0.32%), peonidin-3-sophoroside-5-glucoside (1.29%), cyanidin-3-*p*-hydroxybenzoyl-sophoroside-5-glucoside (4.07%), peonidin-3-*p*-hydroxybenzoyl-sophoroside-5-glucoside (9.61%), cyanidin-3-feruloyl-sophoroside-5-glucoside (1.72%), cyanidin-3-caffeoyl-sophoroside-5-glucoside (1.37%), peonidin-3-feruloyl-sophoroside-5-glucoside (3.69%), cyanidin-3-dicaffeoyl-sophoroside-5-glucoside (2.12%), cyanidin-3-caffeoyl-*p*-hydroxybenzoyl-sophoroside-5-glucoside (11.05%), peonidin-3-caffeoyl-sophoroside-5-glucoside (5.38%), cyanidin-3-caffeoyl-feruloyl-sophoroside-5-glucoside (3.81%), peonidin-3-dicaffeoyl-sophoroside-5-glucoside (3.66%), peonidin-3-caffeoyl-*p*-hydroxybenzoyl-sophoroside-5-glucoside (40.08%), peonidin-3-caffeoyl-feruloyl-sophoroside-5-glucoside (10.85%), peonidin-3-feruloyl-*p*-hydroxybenzoyl-sophoroside-5-glucoside (0.92%)

Roasted PSP: cyanidin-3-sophoroside-5-glucoside (0.83%), peonidin-3-sophoroside-5-glucoside (2.21%), cyanidin-3-*p*-hydroxybenzoyl-sophoroside-5-glucoside (4.86%), peonidin-3-*p*-hydroxybenzoyl-sophoroside-5-glucoside (11.14%), cyanidin-3-feruloyl-sophoroside-5-glucoside (2.15%), cyanidin-3-caffeoyl-sophoroside-5-glucoside (1.66%), peonidin-3-feruloyl-sophoroside-5-glucoside (4.38%), cyanidin-3-dicaffeoyl-sophoroside-5-glucoside (1.82%), cyanidin-3-caffeoyl-*p*-hydroxybenzoyl-sophoroside-5-glucoside (9.76%), peonidin-3-caffeoyl-sophoroside-5-glucoside (6.1%), cyanidin-3-caffeoyl-feruloyl-sophoroside-5-glucoside (3.87%), peonidin-3-dicaffeoyl-sophoroside-5-glucoside (3.67%), peonidin-3-caffeoyl-*p*-hydroxybenzoyl-sophoroside-5-glucoside (36.1%), peonidin-3-caffeoyl-feruloyl-sophoroside-5-glucoside (11.05%), peonidin-3-feruloyl-*p*-hydroxybenzoyl-sophoroside-5-glucoside (0.4%)

Root	Funiu	**Water extract**: peonidin-3-sophoroside-5-glucoside (12.25%), cyanidin-3-*p*-hydroxybenzoyl-sophoroside-5-glucoside (1.92%), peonidin-3-*p*-hydroxybenzoyl-sophoroside-5-glucoside (3.6%), cyanidin-3-caffeoyl-sophoroside-5-glucoside (2.7%), peonidin-3-feruloyl-sophoroside-5-glucoside (0.83%), cyanidin-3-dicaffeoyl-sophoroside-5-glucoside (0.43%), cyanidin-3-caffeoyl-*p*-hydroxybenzoyl-sophoroside-5-glucoside (29.78%), cyanidin-3-caffeoyl-feruloyl-sophoroside-5-glucoside (4.28%), peonidin-3-dicaffeoyl-sophoroside-5-glucoside (8.41%), peonidin-3-feruloyl-*p*-coumaroyl-sophoroside-5-glucoside (7.18%), peonidin-3-caffeoyl-*p*-hydroxybenzoyl-sophoroside-5-glucoside (0.33%), peonidin-3-caffeoyl-feruloyl-sophoroside-5-glucoside (25.64%), peonidin-3-sophoroside-5-glucoside (2.91%)<br>**70% Ethanol extract**: peonidin-3-sophoroside-5-glucoside (1.24%), cyanidin-3-*p*-hydroxybenzoyl-sophoroside-5-glucoside (1.85%), peonidin-3-*p*-hydroxybenzoyl-sophoroside-5-glucoside (1.48%), cyanidin-3-caffeoyl-sophoroside-5-glucoside (4.65%), peonidin-3-feruloyl sophoroside-5-glucoside (0.57%), cyaniding-3-caffeoyl-*p*-hydroxybenzoyl-sophoroside-5-glucoside (21.34%), cyaniding-3-caffeoyl-feruloyl sophoroside-5-glucoside (3.45%), peonidin-3-dicaffeoyl-sophoroside-5-glucoside (11.31%), peonidin-3-feruloyl-*p*-coumaroyl-sophoroside-5-glucoside (9.81%), peonidin-3-caffeoyl-feruloyl-sophoroside-5-glucoside (38.71%), peonidin-3-sophoroside-5-glucoside (5.60%)<br>**High pressure carbon dioxide extract**: peonidin-3-sophoroside-5-glucoside (9.76%), cyanidin-3-*p*-hydroxybenzoyl-sophoroside-5-glucoside (1.15%), peonidin-3-*p*-hydroxybenzoyl-sophoroside-5-glucoside (3.37%), cyanidin-3-caffeoyl-sophoroside-5-glucoside (5.12%), peonidin-3-feruloyl-sophoroside-5-glucoside (0.79%), cyanidin-3-dicaffeoyl-sophoroside-5-glucoside (0.35%), cyanidin-3-caffeoyl-*p*-hydroxybenzoyl-sophoroside-5-glucoside (32.24%), cyanidin-3-caffeoyl-feruloyl-sophoroside-5-glucoside (4.02%), peonidin-3-dicaffeoyl-sophoroside-5-glucoside (8.44%), peonidin-3-feruloyl-*p*-coumaroyl-sophoroside-5-glucoside (6.68%), peonidin-3-caffeoyl-*p*-hydroxybenzoyl-sophoroside-5-glucoside (0.46%), peonidin-3-caffeoyl-feruloyl-sophoroside-5-glucoside (24.03%), peonidin-3-sophoroside-5-glucoside (3.60%)	[58]

Root	Borami, Mokpo 62, Shinzami and Zami	**Borami**: cyanidin-3-sophorside-5-glucoside (0.62%), pelargonidin-3-sophorside-5-glucoside (1.06%), peonidin-3-sophorside-5-glucoside (1.26%), cyanidin-3-*p*-hydroxybenzoyl-sophoroside-5-glucoside (0.28%), cyanidin-3-caffeoyl-sophorside-5-glucoside (0.52%), peonidin-3-*p*-hydroxybenzoyl-sophoroside-5-glucoside (0.74%), peonidin-3-caffeoyl-sophorside-5-glucoside (0.89%), pelargonidin-3-caffeoyl-sophorside-5-glucoside (0.92%), cyanidin-3-*p*-coumaryl-sophorside-5-glucoside (0.2%), cyanidin-3-feruloyl-sophorside-5-glucoside (0.65%), peonidin-3-*p*-coumaryl-sophorside-5-glucoside (0.4%), peonidin-3-feruloyl-sophorside-5-glucoside (1.37%), pelargonidin-3-feruloyl-sophorside-5-glucoside (1.32%,), cyanidin-3-dicaffeoyl-sophorside-5-glucoside (3.96%), cyanidin-3-caffeoyl-sophorside-5-glucoside (4.31%), cyanidin-3-caffeoyl-*p*-hydroxybenzoyl-sophorside-5-glucoside (1.09%), cyanidin-3-caffeoyl-feruloyl-sophoroside-5-glucoside (4.83%), pelargonidin-3-caffeoyl-sophorside-5-glucoside (11.23%), peonidin-3-caffeoyl-sophorside-5-glucoside (12.05%), peonidin-3-dicaffeoyl-sophorside-5-glucoside (11.11%), pelargonidin-3-dicaffeoyl-sophorside-5-glucoside (8.4%), peonidin-3-caffeoyl-*p*-hydroxybenzoyl-sophorside-5-glucoside (3.12%), pelargonidin-3-caffeoyl-*p*-hydroxybenzoyl-sophorside-5-glucoside (1.71%), peonidin-3-caffeoyl-feruloyl-sophoroside-5-glucoside (14.04%), peonidin-3-caffeoyl-*p*-coumaryl-sophorside-5-glucoside (2.2%), pelargonidin-3-caffeoyl-feruloyl-sophoroside-5-glucoside (9.95%), pelargonidin-3-caffeoyl-*p*-coumaryl-sophorside-5-glucoside (1.78%)<br>**Mokpo 62**: cyanidin-3-sophorside-5-glucoside (1.2%), peonidin-3-sophorside-5-glucoside (5.43%), cyanidin-3-*p*-hydroxybenzoyl-sophoroside-5-glucoside (1.3%), peonidin-3-*p*-hydroxybenzoylsoph-5-glucoside (5.35%), peonidin-3-caffeoyl-sophorside-5-glucoside (0.52%), cyanidin-3-feruloyl-sophorside-5-glucoside (0.73%), peonidin-3-feruloyl-sophorside-5-glucoside (2.11%), cyanidin-3-caffeoyl-sophorside-5-glucoside (4.54%), cyanidin-3-caffeoyl-*p*-hydroxybenzoyl-sophorside-5-glucoside (4.65%), cyanidin-3-caffeoyl-feruloyl-sophoroside-5-glucoside (2.35%), pelargonidin-3-caffeoyl-sophorside-5-glucoside (0.57%), peonidin-3-caffeoyl-sophorside-5-glucoside (26.15%), peonidin-3-dicaffeoyl-sophorside-5-glucoside (4.02%), peonidin-3-caffeoyl-*p*-hydroxybenzoyl-sophors	[62]

ide-5-glucoside (28.24%), peonidin-3-caffeoyl-feruloyl-sophoroside-5-glucoside (12.84%)

Shinzami: cyanidin-3-sophorside-5-glucoside (1.02%), peonidin-3-sophorside-5-glucoside (2.7%), cyanidin-3-*p*-hydroxybenzoyl-sophoroside-5-glucoside (4.28%), cyanidin-3-caffeoyl-sophorside-5-glucoside (0.65%), peonidin-3-*p*-hydroxybenzoyl-sophoroside-5-glucoside (11.96%), peonidin-3-caffeoyl sophorside-5-glucoside (1.09%), cyanidin-3-feruloyl-sophorside-5-glucoside (2.05%), peonidin-3-*p*-coumaryl-sophorside-5-glucoside (0.28%), peonidin-3-feruloyl-sophorside-5-glucoside (4.28%), cyanidin-3-dicaffeoyl-sophorside-5-glucoside (2.35%), cyanidin-3-caffeoyl-sophorside-5-glucoside (1.56%), cyanidin-3-caffeoyl-*p*-hydroxybenzoyl-sophorside-5-glucoside (7.94%), cyanidin-3-caffeoyl-feruloyl-sophoroside-5-glucoside (3.32%), peonidin-3-caffeoyl-sophorside-5-glucoside (6.63%), peonidin-3-dicaffeoyl-sophorside-5-glucoside (6.89%), peonidin-3-caffeoyl-*p*-hydroxybenzoyl-sophorside-5-glucoside (32.11%), peonidin-3-caffeoyl-feruloylsoph-5-glucoside (10.9%)

Zami: cyanidin-3-sophorside-5-glucoside (1.27%), peonidin-3-sophorside-5-glucoside (0.55%), cyanidin-3-*p*-hydroxybenzoyl-sophoroside-5-glucoside (1.66%), cyanidin-3-caffeoyl-sophorside-5-glucoside (0.9%), peonidin-3-*p*-hydroxybenzoyl-sophoroside-5-glucoside (0.86%), peonidin-3-caffeoyl-sophorside-5-glucoside (0.3%), cyanidin-3-*p*-coumaryl-sophorside-5-glucoside (0.14%), cyanidin-3-feruloyl-sophorside-5-glucoside (1.66%), peonidin-3-feruloyl-sophorside-5-glucoside (0.56%), cyanidin-3-dicaffeoyl-sophorside-5-glucoside (11.77%), cyanidin-3-caffeoyl-sophorside-5-glucoside (14.77%), cyanidin-3-caffeoyl-*p*-hydroxybenzoyl-sophorside-5-glucoside (18.94%), cyanidin-3-caffeoyl-feruloyl-sophoroside-5-glucoside (17.38%), pelargonidin-3-caffeoyl-sophorside-5-glucoside (0.92%), peonidin-3-caffeoyl-sophorside-5-glucoside (5.4%), peonidin-3-dicaffeoyl-sophorside-5-glucoside (5.02%), pelargonidin-3-dicaffeoyl-sophorside-5-glucoside (0.42%), peonidin-3-caffeoyl-*p*-hydroxybenzoyl-sophorside-5-glucoside (9.25%), peonidin-3-caffeoyl-feruloyl-sophoroside-5-glucoside (7.79%), pelargonidin-3-caffeoyl-*p*-coumaryl-sophorside-5-glucoside (0.45%)

Leaves and roots	Fushu 23, Fushu 25, Ornamental Purple, Yanshu 5, Fushu 9, Fushu 24, Longzishu 4, Longzishu 6 and Fushu 317	**Leaves of Fushu 23**: cyanidin-3-sophoroside-5-glucoside (5.55%), peonidi-3-sophoroside-5-glucoside (7.69%), cyanidin-3-*p*-hydroxybenzoyl-sophoroside-5-glucoside (26.04%), cyanidin-3-caffeylsoph-sophoroside-5-glucoside (2.34%), peonidin-3-*p*-hydroxybenzoyl-sophoroside-5-glucoside (12.98%), peonidin-3-caffeylsoph-sophoroside-5-glucoside (0.54%), cyanidin-3-*p*-coumaryl-sophoroside-5-glucoside (0.6%), cyanidin-3-feruloyl-sophoroside-5-glucoside (1.83%), peonidin-3-*p*-coumaryl-sophoroside-5-glucoside (1.47%), peonidin-3-feruloyl-sophoroside-5-glucoside (0.76%), cyanidin-3-dicaffeyl-sophoroside-5-glucoside (7.43%), cyanidin-3-caffeoyl-*p*-hydroxybenzoyl-sophoroside-5-glucoside (24.65%), cyanidin-3-caffeoyl-*p*-coumaryl-sophoroside-5-glucoside (0.49%), cyanidin-3-caffeoyl-feruloyl-sophoroside-5-glucoside (1.7%), peonidin-3-dicaffeoyl-sophoroside-5-glucoside (0.88%), peonidin-3-caffeoyl-*p*-hydroxybenzoyl-sophoroside-5-glucoside (4.64%), peonidin-3-caffeoyl-*p*-coumaryl-sophoroside-5-glucoside (0.07%), peonidin-3-caffeoyl-feruloyl-sophoroside-5-glucoside (0.32%)<br>**Leaves of Fushu 317**: cyanidin-3-sophoroside-5-glucoside (2.67%), peonidin-3-sophoroside-5-glucoside (4.85%), cyanidin-3-*p*-hydroxybenzoyl-sophoroside-5-glucoside (7.99%), cyanidin-3-caffeylsoph-sophoroside-5-glucoside (8.44%), peonidin-3-*p*-hydroxybenzoyl-sophoroside-5-glucoside (4.73%), peonidin-3-caffeylsoph-sophoroside-5-glucoside (4.01%), cyanidin-3-*p*-coumaryl-sophoroside-5-glucoside (15.47%), cyanidin-3-feruloyl-sophoroside-5-glucoside (3.49%), peonidin-3-*p*-coumaryl-sophoroside-5-glucoside (11.98%), peonidin-3-feruloyl-sophoroside-5-glucoside (3.32%), cyanidin-3-dicaffeyl-sophoroside-5-glucoside (9.28%), cyanidin-3-caffeoyl-*p*-hydroxybenzoyl-sophoroside-5-glucoside (3.82%), cyanidin-3-caffeoyl-*p*-coumaryl-sophoroside-5-glucoside (6.08%), cyanidin-3-caffeoyl-feruloyl-sophoroside-5-glucoside (3.87%), peonidin-3-dicaffeoyl-sophoroside-5-glucoside (4.01%), peonidin-3-caffeoyl-*p*-hydroxybenzoyl-sophoroside-5-glucoside (2%), peonidin-3-caffeoyl-*p*-coumaryl-sophoroside-5-glucoside (2.49%), peonidin-3-caffeoyl-feruloyl-sophoroside-5-glucoside (1.48%)<br>**Leaves of Fushu 25**: cyanidin-3-sophoroside-5-glucoside (2.47%), peonidin-3-sophoroside-5-glucoside (1.52%), cyanidin-3-*p*-hydroxybenzoyl-sophoroside-5-glucoside (2.0	[21]

7%), cyanidin-3-caffeylsoph-sophoroside-5-glucoside (8.29%), peonidin-3-*p*-hydroxybenzoyl-sophoroside-5-glucoside (2.95%), peonidin-3-caffeylsoph-sophoroside-5-glucoside (2.14%), cyanidin-3-*p*-coumaryl-sophoroside-5-glucoside (12.89%), cyanidin-3-feruloyl-sophoroside-5-glucoside (4.68%), peonidin-3-*p*-coumaryl-sophoroside-5-glucoside (16.06%), peonidin-3-feruloyl-sophoroside-5-glucoside (4.45%), cyanidin-3-dicaffeyl-sophoroside-5-glucoside (12.45%), cyanidin-3-caffeoyl-*p*-hydroxybenzoyl-sophoroside-5-glucoside (5.13%), cyanidin-3-caffeoyl-*p*-coumaryl-sophoroside-5-glucoside (8.15%), cyanidin-3-caffeoyl-feruloyl-sophoroside-5-glucoside (2.93%), peonidin-3-dicaffeoyl-sophoroside-5-glucoside (3.69%), peonidin-3-caffeoyl-*p*-hydroxybenzoyl-sophoroside-5-glucoside (4.78%), peonidin-3-caffeoyl-*p*-coumaryl-sophoroside-5-glucoside (3.34%), peonidin-3-caffeoyl-feruloyl-sophoroside-5-glucoside (1.99%)

Leaves of Ornamental Purple: cyanidin-3-sophoroside-5-glucoside (3.26%), peonidin-3-sophoroside-5-glucoside (2.34%), cyanidin-3-*p*-hydroxybenzoyl-sophoroside-5-glucoside (1.11%), cyanidin-3-caffeylsoph-sophoroside-5-glucoside (7.22%), peonidin-3-*p*-hydroxybenzoyl-sophoroside-5-glucoside (5.35%), peonidin-3-caffeylsoph-sophoroside-5-glucoside (4.55%), cyanidin-3-*p*-coumaryl-sophoroside-5-glucoside (17.51%), cyanidin-3-feruloyl-sophoroside-5-glucoside (3.95%), peonidin-3-*p*-coumaryl-sophoroside-5-glucoside (13.56%), peonidin-3-feruloyl-sophoroside-5-glucoside (3.76%), cyanidin-3-dicaffeyl-sophoroside-5-glucoside (10.51%), cyanidin-3-caffeoyl-*p*-hydroxybenzoyl-sophoroside-5-glucoside (4.33%), cyanidin-3-caffeoyl-*p*-coumaryl-sophoroside-5-glucoside (6.88%), cyanidin-3-caffeoyl-feruloyl-sophoroside-5-glucoside (4.38%), peonidin-3-dicaffeoyl-sophoroside-5-glucoside (4.55%), peonidin-3-caffeoyl-*p*-hydroxybenzoyl-sophoroside-5-glucoside (2.26%), peonidin-3-caffeoyl-*p*-coumaryl-sophoroside-5-glucoside (2.82%), peonidin-3-caffeoyl-feruloyl-sophoroside-5-glucoside (1.68%)

Leaves of Yanshu 5: cyanidin-3-sophoroside-5-glucoside (2.78%), peonidin-3-sophoroside-5-glucoside (6.05%), cyanidin-3-*p*-hydroxybenzoyl-sophoroside-5-glucoside (0.94%), cyanidin-3-caffeylsoph-sophoroside-5-glucoside (10.53%), peonidin-3-*p*-hydroxybenzoyl-sophoroside-5-glucoside (4.58%), peonidin-3-caffeylsoph-sophoroside-5-gluco

side (5.01%), cyanidin-3-*p*-coumaryl-sophoroside-5-glucoside (14.96%), cyanidin-3-feruloyl-sophoroside-5-glucoside (4.35%), peonidin-3-*p*-coumaryl-sophoroside-5-glucoside (11.59%), peonidin-3-feruloyl-sophoroside-5-glucoside (4.14%), cyanidin-3-dicaffeyl-sophoroside-5-glucoside (8.98%), cyanidin-3-caffeoyl-*p*-hydroxybenzoyl-sophoroside-5-glucoside (4.76%), cyanidin-3-caffeoyl-*p*-coumaryl-sophoroside-5-glucoside (5.88%), cyanidin-3-caffeoyl-feruloyl-sophoroside-5-glucoside (4.82%), peonidin-3-dicaffeoyl-sophoroside-5-glucoside (3.88%), peonidin-3-caffeoyl-*p*-hydroxybenzoyl-sophoroside-5-glucoside (2.49%), peonidin-3-caffeoyl-*p*-coumaryl-sophoroside-5-glucoside (2.41%), peonidin-3-caffeoyl-feruloyl-sophoroside-5-glucoside (1.85%)

Roots of Fushu 9: cyanidin-3-sophoroside-5-glucoside (3.65%), peonidin-3-sophoroside-5-glucoside (6.23%), cyanidin-3-*p*-hydroxybenzoyl-sophoroside-5-glucoside (3.71%), cyanidin-3-caffeylsoph-sophoroside-5-glucoside (2.22%), peonidin-3-*p*-hydroxybenzoyl-sophoroside-5-glucoside (1.45%), peonidin-3-caffeylsoph-sophoroside-5-glucoside (1.28%), cyanidin-3-feruloyl-sophoroside-5-glucoside (14.82%), peonidin-3-feruloyl-sophoroside-5-glucoside (25.49%), cyanidin-3-dicaffeyl-sophoroside-5-glucoside (1.35%), cyanidin-3-caffeoyl-*p*-hydroxybenzoyl-sophoroside-5-glucoside (4.23%), cyanidin-3-caffeoyl-feruloyl-sophoroside-5-glucoside (11.83%), peonidin-3-dicaffeoyl-sophoroside-5-glucoside (16.9%), peonidin-3-caffeoyl-*p*-hydroxybenzoyl-sophoroside-5-glucoside (1.97%), peonidin-3-caffeoyl-feruloyl-sophoroside-5-glucoside (4.85%)

Roots of Fushu 24: cyanidin-3-sophoroside-5-glucoside (6.25%), peonidin-3-sophoroside-5-glucoside (6.97%), cyanidin-3-*p*-hydroxybenzoyl-sophoroside-5-glucoside (5.92%), cyanidin-3-caffeylsoph-sophoroside-5-glucoside (13.03%), peonidin-3-*p*-hydroxybenzoyl-sophoroside-5-glucoside (24.01%), peonidin-3-caffeylsoph-sophoroside-5-glucoside (1.34%), cyanidin-3-feruloyl-sophoroside-5-glucoside (2.1%), peonidin-3-*p*-coumaryl-sophoroside-5-glucoside (7.2%), peonidin-3-feruloyl-sophoroside-5-glucoside (6.02%), cyanidin-3-dicaffeyl-sophoroside-5-glucoside (5.4%), cyanidin-3-caffeoyl-feruloyl-sophoroside-5-glucoside (1.58%), peonidin-3-dicaffeoyl-sophoroside-5-glucoside (18.44%), peonidin-3-caffeoyl-*p*-hydroxybenzoyl-sophoroside-5-glucoside (1.73%)

Roots of Longzishu 4: cyanidin-3-sophoroside-5-glucoside (0.91%), peonidin-3-sophoroside-5-glucoside (3.6%), cyanidin-3-*p*-hydroxybenzoyl-sophoroside-5-glucoside (2.27%), cyanidin-3-caffeylsoph-sophoroside-5-glucoside (11.32%), peonidin-3-*p*-hydroxybenzoyl-sophoroside-5-glucoside (1.16%), cyanidin-3-feruloyl-sophoroside-5-glucoside (5.98%), peonidin-3-*p*-coumaryl-sophoroside-5-glucoside (1.97%), peonidin-3-feruloyl-sophoroside-5-glucoside (5.5%), cyanidin-3-dicaffeyl-sophoroside-5-glucoside (11.72%), cyanidin-3-caffeoyl-*p*-hydroxybenzoyl-sophoroside-5-glucoside (2.13%), cyanidin-3-caffeoyl-feruloyl-sophoroside-5-glucoside (12.15%), peonidin-3-dicaffeoyl-sophoroside-5-glucoside (28.56%), peonidin-3-caffeoyl-*p*-hydroxybenzoyl-sophoroside-5-glucoside (12.74%)

Roots of Longzishu 6: cyanidin-3-sophoroside-5-glucoside (1.26%), peonidin-3-sophoroside-5-glucoside (10.72%), cyanidin-3-*p*-hydroxybenzoyl-sophoroside-5-glucoside (4.93%), cyanidin-3-caffeylsoph-sophoroside-5-glucoside (10.92%), peonidin-3-*p*-hydroxybenzoyl-sophoroside-5-glucoside (1.92%), peonidin-3-caffeylsoph-sophoroside-5-glucoside (1.43%), cyanidin-3-feruloyl-sophoroside-5-glucoside (7.54%), peonidin-3-*p*-coumaryl-sophoroside-5-glucoside (3.31%), peonidin-3-feruloyl-sophoroside-5-glucoside (24.02%), cyanidin-3-dicaffeyl-sophoroside-5-glucoside (5.5%), cyanidin-3-caffeoyl-*p*-hydroxybenzoyl-sophoroside-5-glucoside (1.56%), cyanidin-3-caffeoyl-feruloyl-sophoroside-5-glucoside (1.47%), peonidin-3-dicaffeoyl-sophoroside-5-glucoside (24.77%), peonidin-3-caffeoyl-*p*-hydroxybenzoyl-sophoroside-5-glucoside (0.63%)

Roots of Fushu 317: cyanidin-3-sophoroside-5-glucoside (4.16%), peonidin-3-sophoroside-5-glucoside (5.69%), cyanidin-3-*p*-hydroxybenzoyl-sophoroside-5-glucoside (11.14%), cyanidin-3-caffeylsoph-sophoroside-5-glucoside (1.98%), peonidin-3-*p*-hydroxybenzoyl-sophoroside-5-glucoside (13.38%), peonidin-3-caffeylsoph-sophoroside-5-glucoside (1.32%), cyanidin-3-feruloyl-sophoroside-5-glucoside (1.12%), peonidin-3-*p*-coumaryl-sophoroside-5-glucoside (5.07%), peonidin-3-feruloyl-sophoroside-5-glucoside (1.71%), cyanidin-3-dicaffeyl-sophoroside-5-glucoside (4.12%), cyanidin-3-caffeoyl-*p*-hydroxybenzoyl-sophoroside-5-glucoside (3.19%), cyanidin-3-caffeoyl-feruloyl-sophor

		oside-5-glucoside (8.14%), peonidin-3-dicaffeoyl-sophoroside-5-glucoside (3.67%), peonidin-3-caffeoyl-*p*-hydroxybenzoyl-sophoroside-5-glucoside (16.5%), peonidin-3-caffeoyl-*p*-coumaryl-sophoroside-5-glucoside (18.01%), peonidin-3-caffeoyl-feruloyl-sophoroside-5-glucoside (0.81%)	
Root	Yan 176	Cyanidin-caffeoy-fumaroy-sophoroside-3-glucoside, peonidin-caffeoyl-hydroxybenzoyl-3-glucoside, peonidin-caffeoyl-sophoroside-3-glucoside, peonidin-caffeoyl-fumaroyl-sophorosid-3-glucoside	[32]
Root		Cyanidin-3-sophoroside-5-glucoside, peonidin-3-sophoroside-5-glucoside, cyanidin-3-*p*-hydroxybenzoyl-sophoroside-5-glucoside, peonidin-3-*p*-hydroxybenzoyl-sophoroside-5-glucoside, cyanidin-3-feruloyl-sophoroside-5-glucoside, peonidin-3-feruloyl-sophoroside-5-glucoside, cyanidin-3-caffeoyl-feruloyl-sophoroside-5-glucoside, peonidin-3-caffeoyl-*p*-hydroxybenzoyl-sophoroside-5-glucoside, peonidin-3-caffeoyl-feruloyl-sophoroside-5-glucoside	[52]
Root	Eshu 8	Cyanidin-3-dicaffeoyl-sophoroside-5-glucoside, cyanidin-3-caffeoyl-*p*-hydroxybenzoyl-sophoroside-5-glucoside, cyanidin-3-caffeoyl-feruloyl-sophoroside-5-glucoside, peonidin-3-caffeoyl-sophoroside-5-glucoside, peonidin-3-dicaffeoyl-sophoroside-5-glucoside, peonidin-3-caffeoyl-*p*-hydroxybenzoyl-sophoroside-5-glucoside, peonidin-3-caffeoyl-feruloyl-sophoroside-5-glucoside	[71]
Root	Chiran Murasaki, Tanegashima Murasaki, Naka Murasaki and Purple sweet	**Chiran Murasaki**: cyanidin-3-sophoroside-5-glucoside (3.9%), peonidin-3-sophoroside-5-glucoside (12.2%), cyanidin-3-caffeoyl-sophoroside-5-glucoside (9.4%), cyanidin-3-caffeoyl-feruloyl-sophoroside-5-glucoside (6.5%), peonidin-3-dicaffeoyl-sophoroside-5-glucoside (5.7%), peonidin-3-caffeoyl-sophoroside-5-glucoside/peonidin-3-caffeoyl-*p*-hydroxybenzoyl-sophoroside-5-glucoside (44.8%), peonidin-3-caffeoyl-feruloyl-sophoroside-5-glucoside (17.5%)<br>**Tanegashima Murasaki**: cyanidin-3-sophoroside-5-glucoside (11.6%), cyanidin-3-dicaffeoyl-sophoroside-5-glucoside (0.8%), cyanidin-3-caffeoyl-sophoroside-5-glucoside	[74]

		(45.5%), cyanidin-3-caffeoyl-feruloyl-sophoroside-5-glucoside (27.9%), peonidin-3-caffeoyl-sophoroside)-5-glucoside/peonidin-3-caffeoyl-*p*-hydroxybenzoyl-sophoroside-5-glucoside (4.2%),<br>**Naka Murasaki**: cyanidin-3-sophoroside-5-glucoside (14.6%), cyanidin-3-dicaffeoyl-sophoroside-5-glucoside (10.2%), cyanidin-3-caffeoyl-*p*-hydroxybenzoyl-sophoroside-5-glucoside (13.1%), cyanidin-3-caffeoyl-sophoroside-5-glucoside (40%), cyanidin-3-caffeoyl-feruloyl-sophoroside-5-glucoside (12.8%), peonidin-3-caffeoyl-sophoroside-5-glucoside/peonidin-3-caffeoyl-*p*-hydroxybenzoyl-sophoroside-5-glucoside (9.3%),<br>**Purple sweet**: cyanidin-3-sophoroside-5-glucoside (7.9%), peonidin-3-sophoroside-5-glucoside (33.8%), cyanidin-3-caffeoyl-sophoroside-5-glucoside (4.5%), cyanidin-3-caffeoyl-feruloyl-sophoroside-5-glucoside (5.7%), peonidin-3-dicaffeoyl-sophoroside-5-glucoside (10.2%), peonidin-3-caffeoyl-sophoroside-5-glucoside/peonidin-3-caffeoyl-*p*-hydroxybenzoyl-sophoroside-5-glucoside (29.1%), peonidin-3-caffeoyl-feruloyl-sophoroside-5-glucoside (8.8%)	
Root		Peonidin-3-*p*-hydroxybenzoyl-sophoroside-5-glucoside (10.4%), peonidin-3-feruloylsophoroside-5-glucoside (4.73%), cyanidin-3-caffeoyl-*p*-hydroxybenzoyl-sophoroside-5-glucoside (3.74%), peonidin-3-caffeoyl-sophoroside-5-glucoside (26.01%), peonidin-dicaffeoyl-sophoroside-5-glucoside (9.79%), peonidin-3-caffeoyl-*p*-hydroxybenzoyl-sophoroside-5-glucoside (29.43%), peonidin-caffeoyl-feruloyl-sophoroside-5-glucoside (15.9%)	[44]
Leaves and roots	P40	**Leaves of P40**: cyanidin-3-sophoroside-5-glucoside (10%), peonidin-3-sophoroside-5-glucoside (2.11%), cyanidin-3-*p*-hydroxybenzoyl-sophoreside-5-glucoside (7.11%), cyanidin-3-caffeoyl-sophoroside-5-glucoside (8.68%), peonidin-3-*p*-hydroxybenzoyl-sophoroside-5-glucoside (2.63%,), peonidin-3-caffeoyl-sopheroside-5-glucoside (2.63%), cyanidin-3-feruloyl-sophoroside-5-glucoside (2.89%), cyanidin-3-dicaffeoyl-sophoroside-5-glucoside (30.53%), cyanidin-3-caffeoyl-phydroxybenzoyl-sophoroside-5-glucoside (25.79%), cyanidin-3-caffeoyl-feruloyl-sophoroside-5-glucoside (5%), peonidin-3-dicaffeoyl-sophoroside-5-glueoside (6.05%), peonidin-3-caffeoyl-phydroxybenzoyl-sophores	[65]

		ide-5-glucoside (3.42%), peonidin-3-caffeoyl-feruloyl-sophoroside-5-glucoside (2.63%), peonidin-3-caffeoyl-*p*-coumaryl-sophoroside-5-glucoside (2.89%)<br>**Roots of P40**: cyanidin-3-sophoroside-5-glucoside (2.38%), peonidin-3-sophoroside-5-glucoside (0.4%), cyanidin-3-*p*-hydroxybenzoyl-sophoreside-5-glucoside (4.62%), cyanidin-3-caffeoyl-sophoroside-5-glucoside (1.37%), peonidin-3-*p*-hydroxybenzoyl-sophoroside-5-glucoside (1.01%), peonidin-3-caffeoyl-sopheroside-5-glucoside (0.36%), cyanidin-3-feruloyl-sophoroside-5-glucoside (2.27%), cyanidin-3-dicaffeoyl-sophoroside-5-glucoside (11.31%), cyanidin-3-caffeoyl-phydroxybenzoyl-sophoroside-5-glucoside (43.27%), cyanidin-3-caffeoyl-feruloyl-sophoroside-5-glucoside (14.33%), peonidin-3-dicaffeoyl-sophoroside-5-glueoside (2.91%), peonidin-3-caffeoyl-phydroxybenzoyl-sophoreside-5-glucoside (12.37%), peonidin-3-caffeoyl-feruloyl-sophoroside-5-glucoside (2.63%), peonidin-3-caffeoyl-*p*-coumaryl-sophoroside-5-glucoside (0.45%)	
Root		Cyanidin-3-sophoroside-5-glucoside (4.41%), peonidin-3-sophoroside-5-glucoside (25.92%), cyanidin-3-*p*-hydroxybenzoyl-sophoroside-5-glucoside (2.94%), peonidin-3-*p*-hydroxybenzoyl-sophoroside-5-glucoside (9.41%), peonidin-3-*p*-hydroxybenzoyl-sophoroside-5-glucoside (3.07%), peonidin-3-feruloyl-sophoroside-5-glucoside (8.36%), cyanidin-3-caffeoyl-sophoroside-5-glucoside (3.61%), cyanidin-3-caffeoyl-*p*-hydroxybenzoyl-sophoroside-5-glucoside (1.92%), cyanidin-3-caffeoyl-feruloyl-sophoroside-5-glucoside (2.06%), peonidin-3-caffeoyl-sophoroside-5-glucoside (18.01%), peonidin-3-caffeoyl-*p*-hydroxybenzoyl-sophoroside-5-glucoside (8.6%), peonidin-3-caffeoyl-feruloyl-sophoroside-5-glucoside (7.76%)	[75]
Root	Yamagawamurasaki	Cyanidin-3-*p*-hydroxybenzoyl-caffeyl-glucopyranoside-5-glucoside, cyanidin-3-dicaffeyl-glucopyranoside-5-glucoside, cyanidin-3-ferulyl-caffeyl-glucopyranosid-5-glucoside, peonidin-3-dicaffeyl-glucopyranoside-5-glucoside, peonidin-3-*p*-hydroxybenzoyl-caffeyl-glucopyranoside-5-glucoside, peonidin-3-feruly1-caffey1-glucopyranoside-5-glucoside	[76]

Root	Stokes Purple, Okinawa and NC 415 clone	**Stokes Purple**: cyanidin-3-sophoroside-5-glucoside (1.48%), peonidin-3-sophoroside-5-glucoside (2.52%), cyanidin-3-*p*-hydroxybenzoyl-sophoroside-5-glucoside (1.48%), peonidin-3-*p*-hydroxybenzoyl-sophoroside-5-glucoside (5%), cyanidin-3-feruloyl-sophoroside-5-glucoside (0.96%), cyanidin-3-caffeoyl-sophoroside-5-glucoside (8.95%), cyanidin-3-dicaffeoyl-sophoroside-5-glucoside (1.69%), peonidin-3-caffeoyl-sophoroside-5-glucoside/cyanidin-3-caffeoyl-*p*-hydroxybenzoyl-sophoroside-5-glucoside (26.09%), cyanidin-3-caffeoyl-feruloyl-sophoroside-5-glucoside (3.86%), peonidin-dicaffeoyl-sophoroside-5-glucoside (2.59%), peonidin-3-caffeoyl-*p*-hydroxybenzoyl-sophoroside-5-glucoside (33.44%), peonidin-caffeoyl-feruloyl-sophoroside-5-glucoside (8.54%)<br>**Okinawa**: cyanidin-3-sophoroside-5-glucoside (8.37%), cyanidin-3-caffeoyl-sophoroside-5-glucoside (2.9%), cyanidin-3-feruloyl-sophoroside-5-glucoside (3.46%), cyanidin-3-caffeoyl-sophoroside-5-glucoside (32.51%), cyanidin-3-dicaffeoyl-sophoroside-5-glucoside (13.38%), peonidin-3-caffeoyl-sophoroside-5-glucoside/cyanidin-3-caffeoyl-*p*-hydroxybenzoyl-sophoroside-5-glucoside (6.83%), cyanidin-3-caffeoyl-feruloyl-sophoroside-5-glucoside (29.76%), cyanidin-3-caffeoyl-*p*-coumaryl-sophoroside-5-glucoside (1.37%), peonidin-3-caffeoyl-*p*-hydroxybenzoyl-sophoroside-5-glucoside (0.49%), peonidin-caffeoyl-feruloyl-sophoroside-5-glucoside (0.92%)<br>**NC 415 clone**: cyanidin-3-*p*-hydroxybenzoyl-sophoroside-5-glucoside (2.74%), peonidin-3-*p*-hydroxybenzoyl-sophoroside-5-glucoside (4.28%), cyanidin-3-feruloyl-sophoroside-5-glucoside (0.67%), cyanidin-3-caffeoyl-sophoroside-5-glucoside (7.62%), cyanidin-3-dicaffeoyl-sophoroside-5-glucoside (3.91%), peonidin-3-caffeoyl-sophoroside-5-glucoside/cyanidin-3-caffeoyl-*p*-hydroxybenzoyl-sophoroside-5-glucoside (31.97%), cyanidin-3-caffeoyl-feruloyl-sophoroside-5-glucoside (3.24%), peonidin-dicaffeoyl-sophoroside-5-glucoside (4.57%), peonidin-3-caffeoyl-*p*-hydroxybenzoyl-sophoroside-5-glucoside (34.76%), peonidin-caffeoyl-feruloyl-sophoroside-5-glucoside (4.17%)	[27]

Root	P40	**Raw PSP**: cyanidin-3-sophoroside-5-glucoside (8.57%), cyanidin-3-*p*-hydroxybenzoyl-sophoroside-5-glucoside (8.71%), cyanidin-3-caffeoyl-sophoroside-5-glucoside (4.18%), peonidin-3-*p*-hydroxybenzoyl-sophoroside-5-glucoside (1.38%), cyanidin-3-feruloyl-sophoroside-5-glucoside (6.81%), peonidin-3-feruloyl-sophoroside-5-glucoside (2.06%), cyanidin-3-dicaffeoyl-sophoroside-5-glucoside (9.86%), cyanidin-3-caffeoyl-*p*-hydroxybenzoyl-sophoroside-5-glucoside (16.54%), peonidin-3-caffeoyl-sophoroside-5-glucoside (19.77%), cyanidin-3-caffeoyl-feruloyl-sophoroside-5-glucoside (12.19%), peonidin-3-caffeoyl-*p*-hydroxybenzoyl-sophoroside-5-glucoside (8.34%), peonidin-3-caffeoyl-feruloyl-sophoroside-5-glucoside (1.58%)<br>**Baked PSP**: cyanidin-3-sophoroside-5-glucoside (9.36%), cyanidin-3-*p*-hydroxybenzoyl-sophoroside-5-glucoside (15.12%), cyanidin-3-caffeoyl-sophoroside-5-glucoside (6.06%), peonidin-3-*p*-hydroxybenzoyl-sophoroside-5-glucoside (2.76%), cyanidin-3-feruloyl-sophoroside-5-glucoside (9.21%), peonidin-3-feruloyl-sophoroside-5-glucoside (2.15%), cyanidin-3-dicaffeoyl-sophoroside-5-glucoside (7.44%), cyanidin-3-caffeoyl-*p*-hydroxybenzoyl-sophoroside-5-glucoside (15.04%), peonidin-3-caffeoyl-sophoroside-5-glucoside (12.82%), cyanidin-3-caffeoyl-feruloyl-sophoroside-5-glucoside (10.28%), peonidin-3-caffeoyl-*p*-hydroxybenzoyl-sophoroside-5-glucoside (8.37%), peonidin-3-caffeoyl-feruloyl-sophoroside-5-glucoside (1.53%)<br>**Steamed PSP**: cyanidin-3-sophoroside-5-glucoside (19.24%), cyanidin-3-*p*-hydroxybenzoyl-sophoroside-5-glucoside (20.95%), cyanidin-3-caffeoyl-sophoroside-5-glucoside (8.1%), peonidin-3-*p*-hydroxybenzoyl-sophoroside-5-glucoside (2.57%), cyanidin-3-feruloyl-sophoroside-5-glucoside (18.38%), peonidin-3-feruloyl-sophoroside-5-glucoside (1.09%), cyanidin-3-dicaffeoyl-sophoroside-5-glucoside (3.97%), cyanidin-3-caffeoyl-*p*-hydroxybenzoyl-sophoroside-5-glucoside (4.52%), peonidin-3-caffeoyl-sophoroside-5-glucoside (8.33%), cyanidin-3-caffeoyl-feruloyl-sophoroside-5-glucoside (7.40%), peonidin-3-caffeoyl-*p*-hydroxybenzoyl-sophoroside-5-glucoside (4.44%), peonidin-3-caffeoyl-feruloyl-sophoroside-5-glucoside (1.01%)<br>**Micro-wave PSP**: cyanidin-3-sophoroside-5-glucoside (14.43%), cyanidin-3-*p*-hydrox	[28]

ybenzoyl-sophoroside-5-glucoside (36.24%), cyanidin-3-caffeoyl-sophoroside-5-glucoside (6.67%), peonidin-3-*p*-hydroxybenzoyl-sophoroside-5-glucoside (6.82%), cyanidin-3-feruloyl-sophoroside-5-glucoside (11.22%), peonidin-3-feruloyl-sophoroside-5-glucoside (1.18%), cyanidin-3-dicaffeoyl-sophoroside-5-glucoside (2.59%), cyanidin-3-caffeoyl-*p*-hydroxybenzoyl-sophoroside-5-glucoside (6.51%), peonidin-3-caffeoyl-sophoroside-5-glucoside (5.1%), cyanidin-3-caffeoyl-feruloyl-sophoroside-5-glucoside (3.76%), peonidin-3-caffeoyl-*p*-hydroxybenzoyl-sophoroside-5-glucoside (4.71%), peonidin-3-caffeoyl-feruloyl-sophoroside-5-glucoside (0.78%)

Pressured cook PSP: cyanidin-3-sophoroside-5-glucoside (15.71%), cyanidin-3-*p*-hydroxybenzoyl-sophoroside-5-glucoside (21.63%), cyanidin-3-caffeoyl-sophoroside-5-glucoside (7.04%), peonidin-3-*p*-hydroxybenzoyl-sophoroside-5-glucoside (2.58%), cyanidin-3-feruloyl-sophoroside-5-glucoside (16.05%), peonidin-3-feruloyl-sophoroside-5-glucoside (1.37%), cyanidin-3-dicaffeoyl-sophoroside-5-glucoside (4.46%), cyanidin-3-caffeoyl-*p*-hydroxybenzoyl-sophoroside-5-glucoside (7.64%), peonidin-3-caffeoyl-sophoroside-5-glucoside (9.1%), cyanidin-3-caffeoyl-feruloyl-sophoroside-5-glucoside (8.41%), peonidin-3-caffeoyl-*p*-hydroxybenzoyl-sophoroside-5-glucoside (4.89%), peonidin-3-caffeoyl-feruloyl-sophoroside-5-glucoside (1.12%)

Fried PSP: cyanidin-3-sophoroside-5-glucoside (11.42%), cyanidin-3-*p*-hydroxybenzoyl-sophoroside-5-glucoside (16.02%), cyanidin-3-caffeoyl-sophoroside-5-glucoside (5.83%), peonidin-3-*p*-hydroxybenzoyl-sophoroside-5-glucoside (1.64%), cyanidin-3-feruloyl-sophoroside-5-glucoside (10.02%), peonidin-3-feruloyl-sophoroside-5-glucoside (1.89%), cyanidin-3-dicaffeoyl-sophoroside-5-glucoside (8.22%), cyanidin-3-caffeoyl-*p*-hydroxybenzoyl-sophoroside-5-glucoside (14.13%), peonidin-3-caffeoyl-sophoroside-5-glucoside (12.33%), cyanidin-3-caffeoyl-feruloyl-sophoroside-5-glucoside (11.42%), peonidin-3-caffeoyl-*p*-hydroxybenzoyl-sophoroside-5-glucoside (5.92%), peonidin-3-caffeoyl-feruloyl-sophoroside-5-glucoside (1.23%)

Leave and root	Bhu Krishna	**Leaves of Bhu Krishna**: cyanidin-3-*p*-hydroxybenzoyl-sophoroside-5-glucoside (1.2%), peonidin-3-*p*-hydroxybenzoyl-sophoroside-5-glucoside (1.68%), cyanidin-3-dicaffeoyl-sophoroside-5-glucoside (8.5%), cyanidin-3-caffeoyl-*p*-hydroxybenzoyl-sophoroside-5-glucoside (31.1%), cyanidin-3-caffeoyl-feruolyl-sophoroside-5-glucoside (1.8%), peonidin-3-dicaffeoyl-sophoroside-5-glucoside (3.13%), peonidin-3-caffeoyl-sophoroside-5-glucoside (10.35%), peonidin-3-caffeoyl-*p*-hydroxybenzoyl-sophoroside-5-glucoside (40.02%), peonidin-3-caffeoyl-feruolyl-sophoroside-5-glucoside (2.22%)<br>**Roots of Bhu Krishna**: cyanidin-3-*p*-hydroxybenzoyl-sophoroside-5-glucoside (0.74%), peonidin-3-*p*-hydroxybenzoyl-sophoroside-5-glucoside (1.75%), cyanidin-3-dicaffeoyl-sophoroside-5-glucoside (4.97%), cyanidin-3-caffeoyl-*p*-hydroxybenzoyl-sophoroside-5-glucoside (8.54%), cyanidin-3-caffeoyl-feruolyl-sophoroside-5-glucoside (7.49%), peonidin-3-dicaffeoyl-sophoroside-5-glucoside (20.24%), peonidin-3-caffeoyl-sophoroside-5-glucoside (6.22%), peonidin-3-caffeoyl-*p*-hydroxybenzoyl-sophoroside-5-glucoside (32.24%), peonidin-3-caffeoyl-feruolyl-sophoroside-5-glucoside (17.8%)	[31]
